# Exploring optimal Taxol® CYP725A4 activity in *Saccharomyces cerevisiae*

**DOI:** 10.1186/s12934-022-01922-1

**Published:** 2022-09-19

**Authors:** Behnaz Nowrouzi, Liang Lungang, Leonardo Rios-Solis

**Affiliations:** 1 Institute for Bioengineering, School of Engineering, The University of Edinburgh, Edinburgh, EH9 3BF UK; 2 Centre for Synthetic and Systems Biology (SynthSys), The University of Edinburgh, Edinburgh, EH9 3BD UK; 3 School of Natural and Environmental Sciences, Newcastle University, Newcastle upon Tyne, NE1 7RU UK

**Keywords:** Taxol®, CYP725A4, Cytochrome P450 monooxygenase, Taxadiene-5α-ol, *Saccharomyces cerevisiae*, Diterpenoids, Whole-cell biocatalysis

## Abstract

**Background:**

CYP725A4 catalyses the conversion of the first Taxol® precursor, taxadiene, to taxadiene-5α-ol (T5α-ol) and a range of other mono- and di-hydroxylated side products (oxygenated taxanes). Initially known to undergo a radical rebound mechanism, the recent studies have revealed that an intermediate epoxide mediates the formation of the main characterised products of the enzyme, being T5α-ol, 5(12)-oxa-3(11)-cyclotaxane (OCT) and its isomer, 5(11)-oxa-3(11)-cyclotaxane (iso-OCT) as well as taxadienediols. Besides the high side product: main product ratio and the low main product titre, CYP725A4 is also known for its slow enzymatic activity, massively hindering further progress in heterologous production of Taxol® precursors. Therefore, this study aimed to systematically explore the key parameters for improving the regioselectivity and activity of eukaryotic CYP725A4 enzyme in a whole-cell eukaryotic biocatalyst, *Saccharomyces cerevisiae*.

**Results:**

Investigating the impact of CYP725A4 and reductase gene dosages along with construction of self-sufficient proteins with strong prokaryotic reductases showed that a potential uncoupling event accelerates the formation of oxygenated taxane products of this enzyme, particularly the side products OCT and iso-OCT. Due to the harmful effect of uncoupling products and the reactive metabolites on the enzyme, the impact of flavins and irons, existing as prosthetic groups in CYP725A4 and reductase, were examined in both their precursor and ready forms, and to investigate the changes in product distribution. We observed that the flavin adenine dinucleotide improved the diterpenoids titres and biomass accumulation. Hemin was found to decrease the titre of iso-OCT and T5α-ol, without impacting the side product OCT, suggesting the latter being the major product of CYP725A4. The interaction between this iron and the iron precursor, δ-Aminolevulinic acid, seemed to improve the production of these diterpenoids, further denoting that iso-OCT and T5α-ol were the later products. While no direct correlation between cellular-level oxidative stress and oxygenated taxanes was observed, investigating the impact of salt and antioxidant on CYP725A4 further showed the significant drop in OCT titre, highlighting the possibility of enzymatic-level uncoupling event and reactivity as the major mechanism behind the enzyme activity. To characterise the product spectrum and production capacity of CYP725A4 in the absence of cell growth, resting cell assays with optimal neutral pH revealed an array of novel diterpenoids along with higher quantities of characterised diterpenoids and independence of the oxygenated product spectra from the acidity effect. Besides reporting on the full product ranges of CYP725A4 in yeast for the first time, the highest total taxanes of around 361.4 ± 52.4 mg/L including 38.1 ± 8.4 mg/L of T5α-ol was produced herein at a small, 10-mL scale by resting cell assay, where the formation of some novel diterpenoids relied on the prior existence of other diterpenes/diterpenoids as shown by statistical analyses.

**Conclusions:**

This study shows how rational strain engineering combined with an efficient design of experiment approach systematically uncovered the promoting effect of uncoupling for optimising the formation of the early oxygenated taxane precursors of Taxol®. The provided strategies can effectively accelerate the design of more efficient Taxol®-producing yeast strains.

**Supplementary Information:**

The online version contains supplementary material available at 10.1186/s12934-022-01922-1.

## Background

**Fig. 1:**
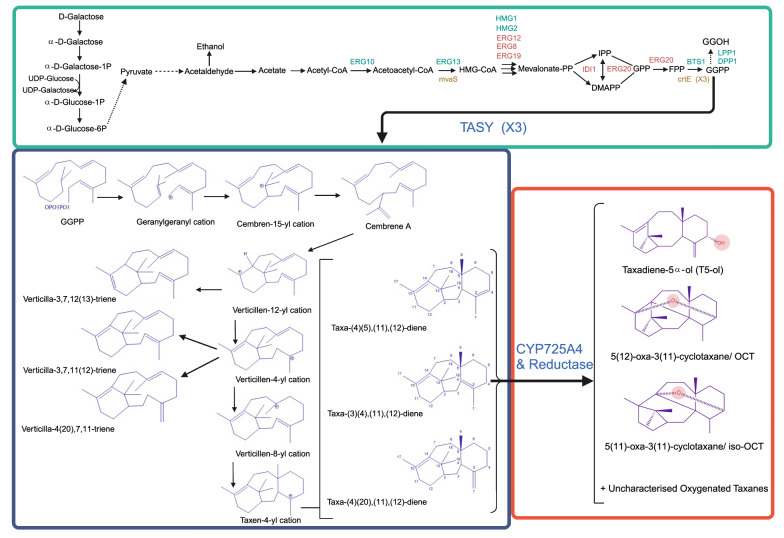
Engineered *S. cerevisiae* cell factory to produce the early Taxol® precursors (taxadiene(s) and oxygenated taxanes), represents the BN6 strain in this study. The overexpressed genes in the mevalonate pathway are highlighted in red and heterologous genes are in olive. In this study, the oxygenated taxanes production starts with galactose induction, following by cyclisation of geranylgeranyl diphosphate (GGPP) to taxadiene and other diterpenes by the diterpene synthase, taxadiene synthase (TASY). CYP725A4 and a cytochrome P450 reductase catalyse the first hydroxylation of the Taxol® pathway, where taxadiene and its isomers are converted to taxadiene-5α-ol (T5α-ol) and other oxygenated taxanes. The heterologous genes include Hydroxymethylglutaryl-CoA synthase (*mvaS*) and acetyl-CoA acetyltransferase/HMG-CoA reductase (*mvaE*) from *Enterococcus faecalis*, and geranylgeranyl diphosphate synthase (*crtE*) from *Xanthophyllomyces dendrorhous*. Three copies of *TASY* with fusion solubility tags are chromosomally integrated in the yeast strains of this study to increase the CYP725A4 substrates (taxadiene and iso-taxadiene) titres [12]. Mevalonate-PP: (R)-5-diphosphomevalonate; HMG-CoA: hydroxymethylglutaryl-CoA; IPP: isopentenyl pyrophosphate; DMAPP: dimethylallyl diphosphate; GPP: geranyl diphosphate; FPP: farnesyl diphosphate; GGPP: geranylgeranyl diphosphate; GGOH: geranylgeraniol. Figure was created with BioRender.com

Taxol® (Paclitaxel), a tricyclic drug originally extracted from *Taxus brevifolia* [1], is one of the most effective anti-cancer drugs in the world [2]. The current commercial routes for production of this valuable drug suffer from being not eco-friendly, heterogenous and low yield, whereby seeking alternative green approaches are deemed essential. In this regard, several microbial and plant-based systems including *Escherichia coli* [3–9], *Saccharomyces cerevisiae* [10–17], *Nicotiana benthamiana* [18, 19], *Nicotiana sylvestris* [20], *Solanum lycopersicum* [21, 22], *Yarrowia lipolytica* [15], *Physcomitrella patens* [23], *Bacillus subtilis* [24], *Arabidopsis thaliana* [25] or co-culture systems [26], have been used to produce the early precursors in Taxol® biosynthetic pathway. However, efforts for introducing the whole biosynthetic pathway have largely been unsuccessful. While the reason can be partially attributed to not all reactions being elucidated, the current obstacle is the relatively poor activity of the first cytochrome P450 enzyme, taxadiene-5α-hydroxylase (CYP725A4), in heterologous hosts. Briefly, the pathway starts with formation of the taxane backbone through macrocyclisation of geranylgeranyl diphosphate (GGPP) by a promiscuous diterpene synthase known as taxadiene synthase. Going through a series of carbocationic transformations and intramolecular proton transfers, several deprotonation events lead to formation of dicyclic verticillenes as well as taxa-4(5),11(12)-diene (taxadiene) and its isomers, whereby taxadiene and taxa-4(20),11(12)-diene (iso-taxadiene) remain the major products [7, 14, 27]. Then, CYP725A4 catalyses the oxygenation of taxadiene using cognate reductase (POR) and NADPH as the cofactor to yield taxa-4(20),11(12)-dien-5α-ol (T5α-ol). However, along with T5α-ol, several side diterpenoids, here referred to as oxygenated taxanes, are also formed, among which 5(12)-oxa-3(11)-cyclotaxane (OCT) and 5(11)-oxa-3(11)-cyclotaxane (iso-OCT) have been chemically characterised (Fig. 1). Depending on the experimental conditions, the ratio of T5α-ol: other oxygenated taxanes differ, more in favour of side products, where they can compose up to 85% to 95% of the whole products [7, 14, 15, 20] or in favour of the production of T5α-ol isomer [13, 14]. In contrast, there has been a great progress in producing taxadiene in microbial hosts, reaching maximum 1 g/L in *E. coli* [3] and 129 mg/L in *S. cerevisiae* [12]. To date, Walls et al. [13] have reported the highest T5α-ol and Taxadien-5α-yl-acetate titres, 35 mg/L and 12 mg/L, respectively, using an optimised synthetic medium in a 1 L batch cultivation. The same study also reported a hypothetical isomer to T5α-ol as the main product, with maximum 73 mg/L final concentration, with comparable concentration of OCT and iso-OCT to that of T5α-ol. They justified this by the change in enzyme selectivity using optimal pH control. Edgar et al. [15] also reported that the ratio of main product: side product varies relative to the extraction method and heterologous chassis and Sagwan-Barkdoll and Anterola [7] proposed that the T5α-ol was the minor product in *E. coli*. Therefore, recent studies skipped to investigate the ideal conditions for T5α-ol formation and reported on the total oxygenated taxanes instead [6, 14, 26].

As a result of such discrepancies, different proposals have been discussed on the CYP725A4 mechanism of activity, which have questioned the existence of radical rebound mechanism [28] for CYP725A4 taxadiene metabolism. In this regard, studies by Edgar et al. [15] and Barton et al. [22] have discussed that CYP725A4 relies on an intermediary epoxidation mechanism, for which they used the chemically-synthesised epoxides. In these proposals, upon the epoxide formation and degradation, different oxygenated taxanes are formed. However, the iso-taxadiene conversion to T5α-ol still undergoes the radical rebound mechanism.

Building on previous studies, our study aimed to systematically find the optimal conditions for favourable activity of CYP725A4 in *S. cerevisiae* at both enzymatic and whole-cell levels, which would accelerate the integration of the rest of the P450-dominated Taxol® biosynthetic pathway genes. While exploring the conditions for increasing the main product titre, several lines of evidence clearly showed that the uncoupling reaction by the *Taxus* CYP725A4 and POR interaction potentially mediates the formation of oxygenated taxanes in *S. cerevisiae* and the T5α-ol titre can be likely improved through optimal fusion protein construction.

## Results and discussions

### Gene dosage study

Major studies in expression of *CYP725A4* in microbial chassis have so far focused on modifications to the P450 and cognate reductase sequences for improved solubility and expression (6), tandem P450-reductase expression (14) and constructing P450-reductase chimeras using strong eukaryotic reductases from human, rat liver or *Stevia rebaudiana* and cytochrome b5 [7]. However, it remains necessary to optimise the CYP725A4 functionality in *S. cerevisiae* cell factories and therefore, the first question we sought to answer was to determine the rate-limiting gene through a gene dosage study.

The effect of increased *CYP725A4* or *Taxus POR* gene copies was evaluated in a three-day run where BN1 strain, expressing an extra copy of *CYP725A4*, improved the diterpenoid I concentration significantly (p = 2.5e−02), proposed to be a potential isomer to T5α-ol in our earlier study [14]. Also, BN2, expressing an extra copy of *POR*, showed a significant impact on OCT (p = 8.2e−03), iso-OCT (p = 0.02) and diterpenoid I (p = 6.6e−07) formation. BN3*,* expressing one copy of *CYP725A4* with a low-glucose inducible promoter*,* pHXT7, also showed significant improvement in OCT (p = 0.0001), iso-OCT (p = 0.002), and diterpenoid I formation (p = 1.3e−03). However, factoring in the impact of interaction between the production time and the strain revealed more detailed results. In more detail, the interaction between the strain type and growth time was found to significantly affect the taxadiene (p = 2.6e−08), OCT (p = 0.0015), taxadienediol (dioxygenated taxane; p = 0.003), diterpenoid I (p = 3.9e−08) and iso-taxadiene (p = 7.4e−09) titres. However, this interaction was found insignificant towards the T5α-ol and iso-OCT titres (p = 0.06 and 0.06, respectively).

**Fig. 2:**
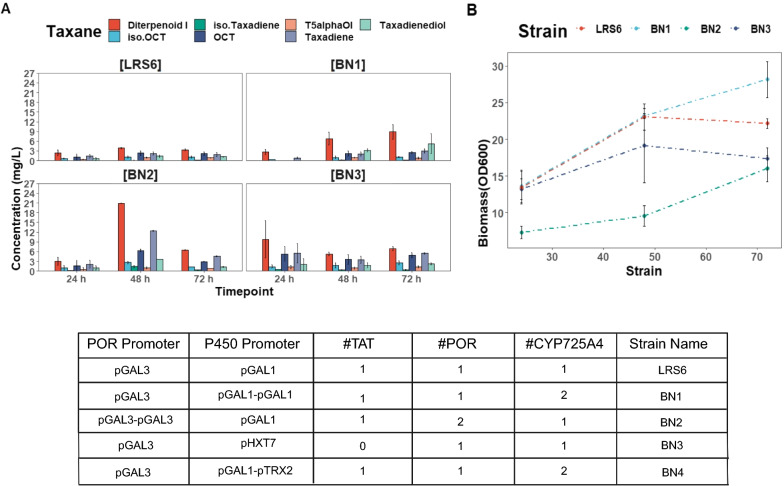
Gene dosage effect on taxanes production. LRS6 and BN1-4 were cultivated in 50 mL of YPG medium in shake flasks for 72 h. **A** Taxanes and **B** biomass curves for LRS6, BN1, BN2 and BN3. BN4 data is not shown as no growth and productivity was observed during the run. The data represents mean ± SD of triplicates. The ‶#″ symbol stands for number. The *CYP725A4* is overexpressed in BN1, BN3 and BN4 and *POR* is overexpressed in BN2. LRS6 is the parent strain to BN1, BN2 and BN4 strains and LRS5 (taxadiene-producing strain) is the parent strain for BN3 strain. Iso.Taxadiene and iso.OCT represent iso-taxadiene and iso-OCT. Figure was created with BioRender.com

As depicted in Fig. 2, doubling the *CYP725A4* gene copy numbers in BN1 did not improve the final T5α-ol in comparison to its parent strain, LRS6. However, OCT concentration was slightly improved by 1.2-fold (2.5 ± 0.3 mg/L, p = 1). Interestingly, diterpenoid I remained the major product in all the new strains with 2.6- and 1.9-fold improvement in its titre in BN1 and BN2, respectively, despite this improvement being insignificant (p = 7.8e−02 and 8.7e−01).

On the other hand, none of the strains showed any enhancement in taxadiene consumption and its titre was increased by 1.5- and 2.4-fold, in BN1 and BN2. Based on the data from Fig. 2, it was clear that the overexpression of *CYP725A4* enzyme neither favoured the taxadiene conversion nor it caused any remarkable increase in T5α-ol production, while it improved the production of side oxygenated taxane products. Furthermore, BN2, with one more copy of *Taxus POR*, showed a decreased final biomass accumulation by 52% (OD_600_: 16 ± 1.7) relative to LRS6. Similar improvement in the side oxygenated taxanes concentrations was initially observed for BN2, however, the final taxadiene concentration in this strain showed 1.5-fold improvement to that of BN1 strain, being 4.6 ± 0.2 mg/L. Despite this, due to the poor performance of BN2 in comparison to BN1, particularly its instability in later experiments and no growth, the next strains were created with focusing on P450 overexpression. In line with our results, the increased *POR* expression also reduced the CYP725A4 oxygenated taxanes concentration in Biggs et al. [6] study using *E. coli*, the phenomenon which authors attributed to resource competition, elevated burden and inefficient use of NADPH. They also noted that increasing *CYP725A4* expression to *POR* resulted in higher accumulation of oxygenated taxanes.

Similar to our previous study [14], we did not detect any acetylated taxane formed due to Taxa-4(20),11(12)-dien-5α-olO-Acetyl Transferase (*TAT*) expression. Despite this, to account for any product changes due to *TAT* expression, BN3 was created with the 1st copy of *CYP725A4* under the control of a different strong constitutive promoter (pHXT7) using taxadiene-producing LRS5. *HXT7* gene is known to be repressed during high glucose availability [29], and since the carbon used in this study was galactose, it was deemed that it would produce a stronger effect on overall taxanes and T5α-ol formation. In comparison to BN1, T5α-ol and OCT titres were further increased by around 1.4-fold and 2.0-fold, respectively, regardless of around 1.8-fold increase in taxadiene titre obtained at 72 h. Despite this, all these changes were found to be insignificant (T5α-ol: adjusted p = 1, OCT: adjusted p = 0.35 and taxadiene: adjusted p = 0.47) compared to BN1 at the end of the cultivation (72 h). However, comparing the oxygenated taxanes production by LRS6 and BN3, showed that despite using different promoters, TAT activity likely led to the consumption of all the major oxygenated taxanes products, with 1.4-, 2.3- and 2.3-fold lower T5α-ol, OCT and iso-OCT titres by LRS6, compared to BN3, without *TAT* expression cassette. Hence, it is possible that the side oxygenated products also play intermediary roles in Taxol® biosynthesis pathway that need to be further investigated using the same promoter and terminator sets for the overexpression. Also, the final biomass accumulation of BN3 was reduced to 17.3 ± 1.5, compared to BN1 and LRS6 with final biomass accumulations of 28.14 ± 2.5 and 22.2 ± 0.7, respectively. Hence, it was hypothesised that potentially the taxanes and/or their producing enzymes were inducing the oxidative stress in the cell, lowering the biomass accumulation.

Using the *TRX2* gene promoter, which has been found to be responsive to hydroperoxide stresses [30], BN4 strain was constructed to integrate the 2nd copy of *CYP725A4*. This strain displayed no growth and therefore, no productivity. Biggs et al. has also separately reported that the higher production of CYP725A4 diterpenoids in *E. coli* resulted in loss of productivity [6]. In their recent follow-up study [31], they attributed this phenomenon to potential membrane stress. It was therefore postulated that the increased abundance of the heterologous products together with the overexpressed P450 were cytotoxic. Furthermore, since the constructed strains did not favour the production of T5α-ol and efficient taxadiene conversion, the approach of multiplexing *POR* and *CYP725A4* was deemed inefficient. The chromatograms of these strains are available in Additional file 1: Fig. S2 and the representative mass spectra are presented in Additional file 1: Fig. S3.

The production of the oxygenated taxanes, other than T5α-ol, could also be viewed to be influenced by the electron coupling and P450-reductase interactions efficiency, growth-based and/or no-growth-involved endogenous reactions, and P450/reductase enzymatic components. Therefore, in the rest of our study, we examined the accuracy of these hypotheses through a range of strain engineering and biochemical assays.

### Primary screening of self-sufficient strains

Our first hypothesis was rooted in the possibility of inefficient electron coupling and P450-reductase interactions that result in the side products as the electron transfer occurs in two steps [32]. Indeed, the optimal electron transfer is one of the most important parameters for improved P450 activity and decreased cellular toxicity due to the electron waste in reactive oxygen species (ROS) formation [33]. As all the previous studies had only used eukaryotic PORs and ignored the prokaryotic counterparts, we selected the reductase genes from several natural self-sufficient P450 enzymes. Briefly, as indicated, cytochrome P450 enzymes rely on redox partners/reductases to abstract the electron from NADPH, where in eukaryotes they are generally separately expressed [34]. In contrary, where both P450 heme and the diflavin reductase domains are fused to form a single polypeptide, the cytochrome P450s are called self-sufficient [35]. Self-sufficient enzymes are known for their very high turnover number as a result of optimal electron transfer and coupling, and minimal electron leakage [36]. A comprehensive overview of the self-sufficient enzymes have been covered in our previous review [33].

**Fig. 3:**
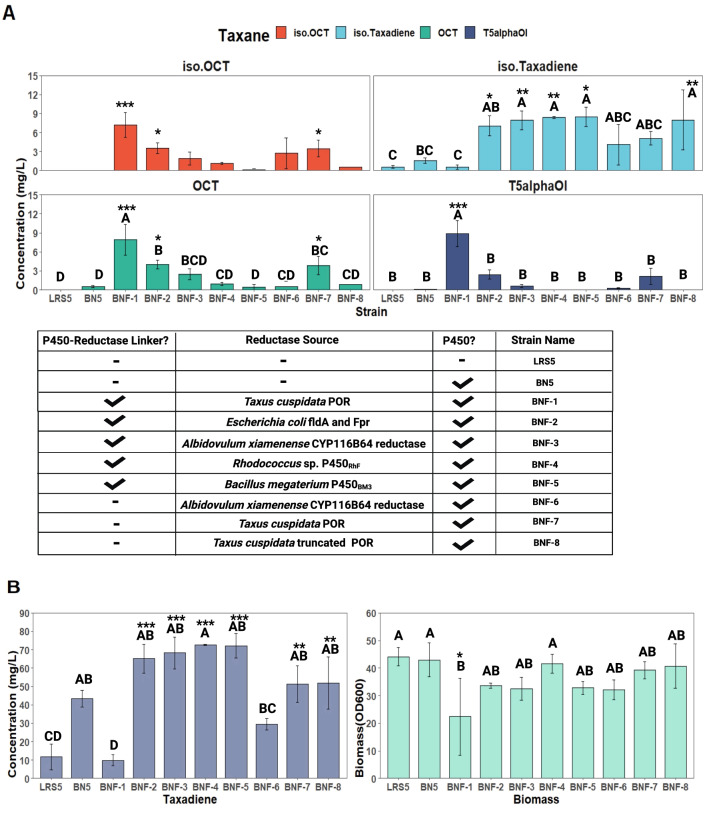
Screening the performance of strains expressing self-sufficient fused *CYP725A4* and reductases (BNF-1-7), self-sufficient fused *CYP725A4* and transmembrane domain-truncated *Taxus cuspidata* reductase (*POR*) (BNF-8), separate *CYP725A4* and *T. cuspidata POR* (BN6), *CYP725A4* only (BN5), and parent taxadiene-producing LRS5 strain. **A** The strains were cultivated in 5 mL of YPG and **A** taxane products and **B** taxadiene and biomass accumulation were measured at the end of the 72 h. Bars represent mean ± SD for triplicates. Tukey's HSD post hoc test shows a measure of difference across the whole strain groups, where different letters indicate significant differences of means (p < 0.05). The asterisks indicate significant differences relative to LRS5 (for taxadiene, iso-taxadiene and OD_600_) and BN5 (for taxanes) by pairwise t-test (***p < 0.001, **p < 0.01, *p < 0.05 adjusted p values)). Iso.Taxadiene, t5alphaOl and iso.OCT represent iso-taxadiene, T5α-ol and iso-OCT. Figure was created with BioRender.com

As a control, a strain expressing *CYP725A4* only (BN5) was also constructed. Interestingly, the strain only expressing the P450 enzyme still produced T5α-ol, OCT and iso-OCT, although at minor quantities of 0.11 ± 0.02, 0.51 ± 0.2 mg/L, and below the limit of quantification detection for iso-OCT, which represented a reduction of 77.4- and 15.4-fold for T5α-ol and OCT, respectively, in comparison to the strain BNF-1, expressing *Taxus POR* (Fig. 3). This denoted that the yeast native redox metabolism contributes to the production of oxygenated taxanes.

As shown in Fig. 3A, interestingly, among the self-sufficient strains, only BNF-1, BNF-2 and BNF-7 showed significant differences to BN5 strain in OCT and iso-OCT production. However, except BNF-1, no other strains depicted any significantly different results to BN5 in terms of T5α-ol formation. Also, taxadiene and iso-taxadiene titre significantly improved in all *CYP725A4*-expressing strains opposed to BNF-1 and LRS5 (Fig. 3B); In comparison to taxadiene-producing strain, LRS5, surprisingly, there was a significant increase in taxadiene production by BNF-2 (adjusted p = 0.002), BNF-3 (adjusted p = 0.001), BNF-4 (adjusted p = 0.0004), BNF-5 (adjusted p = 0.002), BNF-7 (adjusted p = 0.04) and BNF-8 (adjusted p = 0.0012). Similarly, iso-taxadiene was significantly improved by BNF-2, BNF-3, BNF-4, BNF-5 and BNF-8, representing 12.3-, 13.9-, 14.7-, 14.8- and 13.9-fold improvement to that of LRS5. Despite this, the final biomass did not significantly change in all strains, except in BNF-1 with 1.45-fold reduction relative to LRS5. By conducting Tukey's HSD post hoc test, a measure of difference across the whole strain groups were revealed. Only BNF-1, BNF-2 and BNF-7 were grouped as significantly different in terms of OCT production, while BNF-1 stood as the most significant in T5α-ol and iso-OCT production.

Interestingly, OCT remained the major product of CYP725A4 in all strains as illustrated in representative chromatograms in Additional file 1: Fig. S4. While BNF-2 was constructed using non-codon-optimised *E. coli FldA* and *Fpr* genes, these genes have been shown to support the microsomal P450 activity at 1:1 ratio, while being less efficient than *S. cerevisiae* POR [37]. Despite this, the other reason for the relatively poor activity of BNF-2 could be the absence of an effective domain for connecting the *Fpr* and *Fld* for optimal NADPH oxidation. Also, it must be noted that the *E. coli* reductases are neither classified as self-sufficient proteins nor reported to have efficient electron coupling power. On the other hand, solubilising *Taxus* POR through truncating its transmembrane sequence also did not help in oxygenated taxanes production in BNF-8, since BNF-7 with whole *POR* sequence produced 85% more oxygenated taxanes. This result was in line with [38], where they proposed that the soluble (truncated) POR could not interact with the P450 and the impact of artificial linker in P450 activity was highlighted. However, this was also in contrast to Biggs et al. study, where they proposed that decreasing POR lipophilicity enabled better CYP725A4-POR interactions and electron transfer in *E. coli* [6].

Of note, the final biomass accumulations of these self-sufficient strains were not found to be significantly different despite their overall poor performance. However, the taxadiene production was found to be higher by at least 3.9-fold as in BN5 to 6.2-fold in BNF-4 compared to taxadiene-producing LRS5 strain, without significant difference in final biomass accumulation. On the contrary, around 1.2-fold reduction in taxadiene formation was noticed in BNF-1 compared to LRS5. Therefore, BNF-1 expressing *Taxus POR* was found to be the best performing strain as more products were obtained, clearly showing a lower concentration of substrate taxadiene in the end of the experiments. As the linkers for these strains were selected from previous studies [39–43], it can be argued that the homology modelling for *Taxus* CYP725A4-POR proteins pair would give better insights on efficient linker design to optimise the enzymatic activity and the substrate consumption. However, except BNF-1, BNF-2 and BNF-7, the use of linker-containing and linker-free constructs both did not improve the production of the oxygenated taxanes. Also, except BNF-1 (p = 0.008), there was no significant difference between final taxadiene concentration of CYP725A4-expressing strain (BN5) and the self-sufficient strains. Hence, it is more likely that suboptimal expression of prokaryotic reductases with strong coupling capacity [44, 45] caused the suboptimal performance of these strains.

### Tandem expression of *Taxus CYP725A4* and *POR* and the effect of other media on the product spectrum

**Fig. 4:**
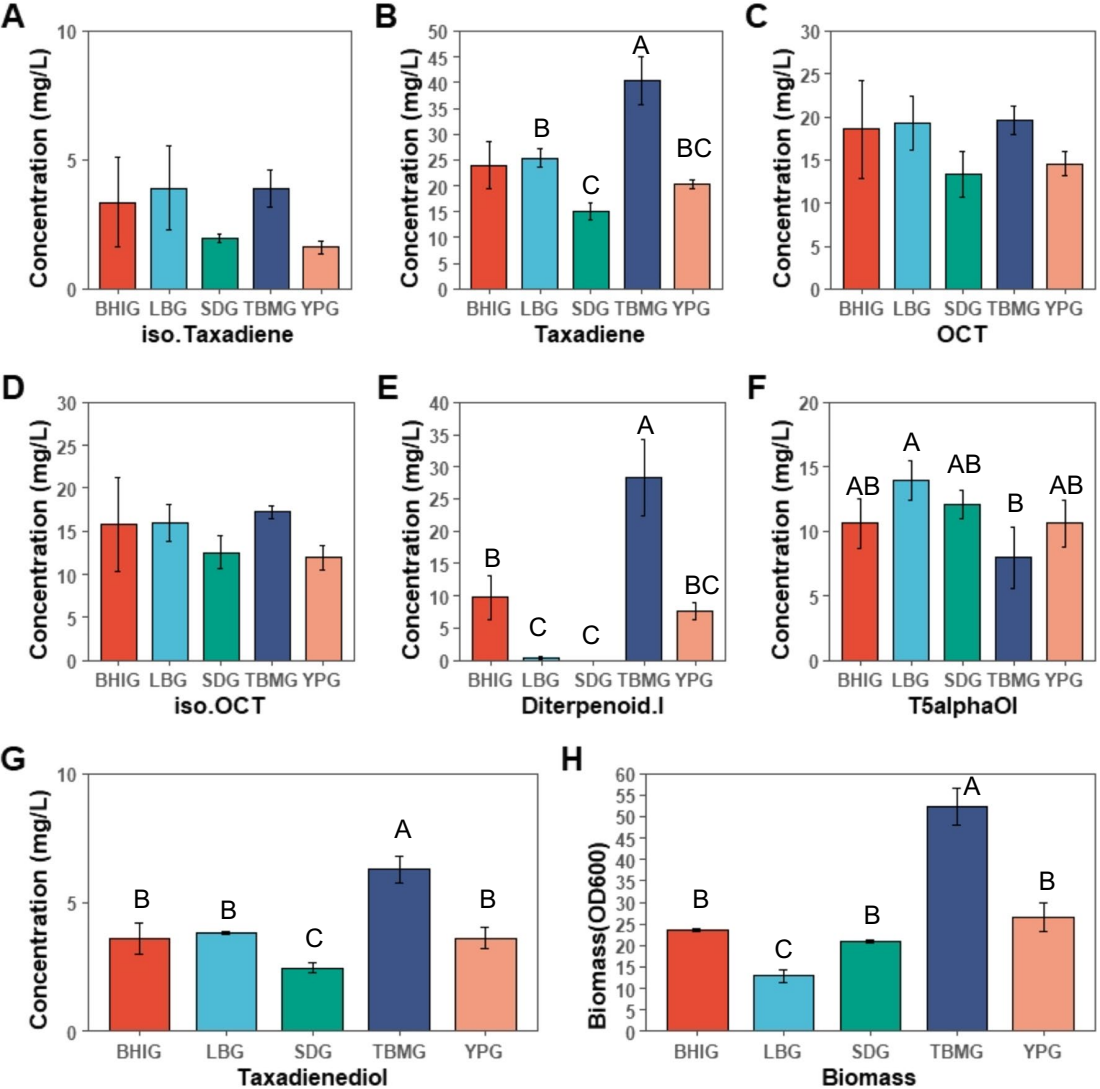
Screening the performance of BN6 strain expressing *Taxus cuspidata CYP725A4* and reductase in tandem. **A**–**G** Taxane distribution and **H** biomass in different media in a 5-mL mini-scale for 72 h. Bars represent mean ± SD for triplicates. Tukey's HSD post hoc test shows a measure of difference across the whole strain groups, where different letters indicate significant differences of means (p < 0.05). The used media were: BHIG: Brain Heart Infusion Broth-2% (w/v) galactose; LBG: LB Broth (Lennox)-2% (w/v) galactose; SDG: Synthetic Defined-2% (w/v); YPG: Yeast Extract Peptone-2% (w/v) galactose; TBMG: Terrific Broth, Modified-2% (w/v) galactose. Iso.Taxadiene and iso.OCT represent iso-taxadiene and iso-OCT. Iso.Taxadiene*,* t5alphaOl, iso.OCT and Diterpenoid.I represent iso-taxadiene*,* T5α-ol, iso-OCT and diterpenoid I. Figure was created with BioRender.com

In the previous section, we showed that the superior coupling capacity of prokaryotic reductase did not favour the CYP75A4 activity in yeast. Therefore, another strain, BN6, was constructed using *Taxus cuspidata* reductase (*POR*). As LRS6 strain was constructed using GAL promoters for both *CYP725A4* and *POR* genes, to minimise any delay in electron transfer, *POR* was expressed under a strong consecutive promoter (pTDH3), while *CYP725A4* was expressed using the strong galactose promoter (pGAL1) for BN6 strain. However, to keep *CYP725A4* expression stronger than that of *POR*, stronger terminator (RPL41Bt) and weaker terminator (ICY2t) [46] were used for *CYP725A4* and *POR*, respectively. In YPG medium, BN6 generated the highest amounts of OCT, iso-OCT and T5α-ol at the final concentrations of 14.6 ± 1.4, 12 ± 1.4 and 10.6 ± 1.8 mg/L, respectively, which represented 1.8-, 1.7-, and 1.2-fold improvement compared to BNF-1, expressing *Taxus POR* fused to *CYP725A4*. However, taxadiene was almost doubled to be 20.3 ± 0.9 mg/L and the final biomass slightly reduced to 26.5 ± 3.2 by the end of 72 h of cultivation (Fig. 4). This denotes the cumulative negative effect of *Taxus* reductase expression and oxygenated taxanes as well as possibly higher allocation of NADPH for non-growth associated tasks in this strain. The higher oxygenated taxanes production compared to self-sufficient strains also denotes that potentially the uncoupling promoted the formation of the oxygenated taxanes. Hence, it is possible that besides the molecular oxygen, the formation of oxygenated taxanes by CYP725A4 was also mediated by other reaction intermediates which acted as the terminal electron and oxygen acceptor(s) [47]. On this matter, the 1.73-fold improved taxadiene titre in BN6 and 3.9-fold in BN5 compared to LRS5 might be due to activation of oxidative stress response [31] by uncoupling, and as a measure to combat the GGPP stress [48]. This proposal is also justified by slightly lower (0.84-fold) taxadiene accumulation in BNF-1 compared to LRS5. Hence, it is plausible to propose that the increased oxygenated taxanes was due to increased taxadiene production, where the P450-POR reaction intermediates induced the higher taxadiene production, without fully converting it to oxygenated taxanes. Then, that can also be explained by the slow uncoupling rate that led to 1.64-fold lower oxygenated taxanes productivity in BNF-1 (0.55 mg/L/OD_600_) compared to BN6 (0.90 mg/L/OD_600_), a phenomenon also reported for other P450-reductase fusions [49]. Thus, it can be concluded that the uncoupling is the promoting agent in the oxygenated taxanes production which occurs efficiently through *Taxus* CYP725A4 and POR interaction. This is also supported by the epoxidation theory [15, 22], where the radical and cationic intermediates favour the formation of epoxides and hydroxides [50]. From another perspective, the presence of POR was the key to increased oxygenated taxanes production, as its electron transfer capability led to 38.1-fold higher oxygenated taxanes titre compared to BN5, only expressing *CYP725A4*. However, as of now, it is unclear how the interaction between CYP725A4 and POR can be optimised to promote the uncoupling for higher oxygenated taxanes production whilst maximising the taxadiene consumption.

Interestingly, the T5α-ol: OCT: iso-OCT ratio remained very similar, 1:0.89:0.81 and 1:1.4:1.1 for BNF-1 and BN6, respectively, by the end of 72-h cultivation in YPG medium. Since uncoupling is an auxiliary mechanism of activity, its effect on product distribution cannot be inferred through in vivo studies. Instead, as results suggest, and due to unknown reasons, T5α-ol is the later product formed when taxadiene consumption is high and possibly the least affected by the decreased uncoupling rate, given the electron transfer to CYP725A4 is established. Consequently, more optimal fusion protein design can be exploited to improve the P450-POR interaction for higher taxadiene conversion and oxygenated taxanes production. Despite this, due to the superior capability of BN6 for higher oxygenated taxanes production, this strain was used for all the other experiments performed later to answer our other hypotheses.

Our second formulated hypothesis was the cumulative effects of endogenous, underground metabolism [51] on the product distribution of the CYP725A4. Our previous study showed a correlation of taxadiene production with biomass accumulation in LRS5 [12]. The latest study by Walls et al. [13] showed that the increased biomass resulted in improved total taxanes concentration. However, a lower dependence of oxygenated taxanes on the biomass proved that the growth and oxygenated taxanes production were not tightly correlated as they reported (Fig. 4). On the other hand, an earlier study by Edgar et al. [15] had illustrated the effect of two rich and minimal media types on the distribution of oxygenated taxanes from CYP725A4 in different microbial systems. Since the potential effect of media type on the product profile was not examined in detail in *S. cerevisiae* yet, we postulated that selecting a variety of microbiology media might be influential in a change in CYP725A4 product spectrum in *S. cerevisiae*.

The results clearly indicated that using more enriched medium like TBMG, while promoting the biomass accumulation of BN6 (Fig. 4H) to 52.3 ± 4.4, almost doubled the titre ratio of OCT+ iso-OCT to T5α-ol. However, using minimal media like SDG and LBG did not result in any difference in this ratio and the use of BHIG medium only increased this figure by around 1.3-fold. One-way ANOVA analysis also revealed that the medium type only had significant effect on taxadiene (p = 2.6e−05), diterpenoid I (p = 3.4e−06), T5α-ol (p = 0.025) and taxadienediol (p = 6.42e−06) titres, and biomass (p = 4.5e−08) (Figs. 4B, E, F, G, H), but no significant impact on iso-taxadiene, OCT and iso-OCT were detected (Figs. 4A, C, D). Tukey post hoc test further clarified that LBG culture had the significantly highest T5α-ol titre (14 ± 1.5 mg/L), while using TBMG medium led to the highest concentration of another potential isomer of T5α-ol, here called diterpenoid I (28.3 ± 5.9 mg/L). Interestingly, diterpenoid I was not found in SDG media (Additional file 1: Fig. S5). In all media, taxadiene was still produced, where TBMG resulted in its maximal production (40.3 ± 4.8 mg/L) and SDG produced the least taxadiene (15 ± 1.6 mg/L). However, there was no significant difference between LBG, BHIG and YPG in terms of taxadiene and taxadienediol production. Also, in terms of biomass accumulation, YPG, BHIG and SDG were grouped together, while LBG resulted in the least biomass accumulation (13 ± 1.5 mg/L). Comparing based on the productivity, the total OCT, iso-OCT and T5α-ol productivity in BHIG, LBG, SDG, TBMG and YPG were 1.9, 3.8, 1.8, 0.9 and 1.4 mg/L/OD_600_, highlighting that the promotion of biomass is not tightly correlated to increased oxygenated taxanes production. Despite this, these ratios changed to 1, 2, 0.7, 0.8 and 0.8 mg/L/OD_600_ for taxadiene, showing that the taxadiene production was coupled to biomass production [12]. As SDG is composed of complete synthetic mixture medium, which is enriched in amino acids, it can be postulated that the cellular NADPH was saved in favour of P450 activity. Supportive of this hypothesis, TBMG which contains a double amount of the yeast extract to tryptone (carbon to nitrogen ratio (C/N):2:1) opposite to YPG (C/N:1:2) promoted the formation of carbon-containing taxanes backbone. However, the lower nitrogen availability potentially directed the flux towards essential cellular tasks like amino acid biosynthesis than heterologous products. Regardless of the type of medium, OCT remained the major product in all media (Fig. 4C; Additional file 1: Fig. S5), and SDG was found superior to YPG for increased oxygenated taxanes production.

### Definitive screening design interpretation

While *S. cerevisiae* was shown capable of producing early oxygenated taxanes, varying the electron transfer partner or reductase gene dosage did not necessarily help with higher CYP725A4 activity as shown in Figs. 2 and 3. Therefore, as the third hypothesis, it was postulated that the other enzymatic parameters might be important to control, while considering that the improvement of P450 expression does not guarantee a parallel improvement in the product titre [52]. Built on our previous results in the gene dosage study, which strengthened the assumption on the promoting effect of uncoupling on oxygenated taxanes formation, it was therefore hypothesised that the induced toxicity might necessitate exogenous sources of cofactors to assist with replenishing the lost resources. Among these factors, the components of the prosthetic groups of both *Taxus* CYP725A4 and reductase proteins, being heme and flavins, respectively, were selected to be supplemented exogenously in the BN6 growth medium.

**Fig. 5:**
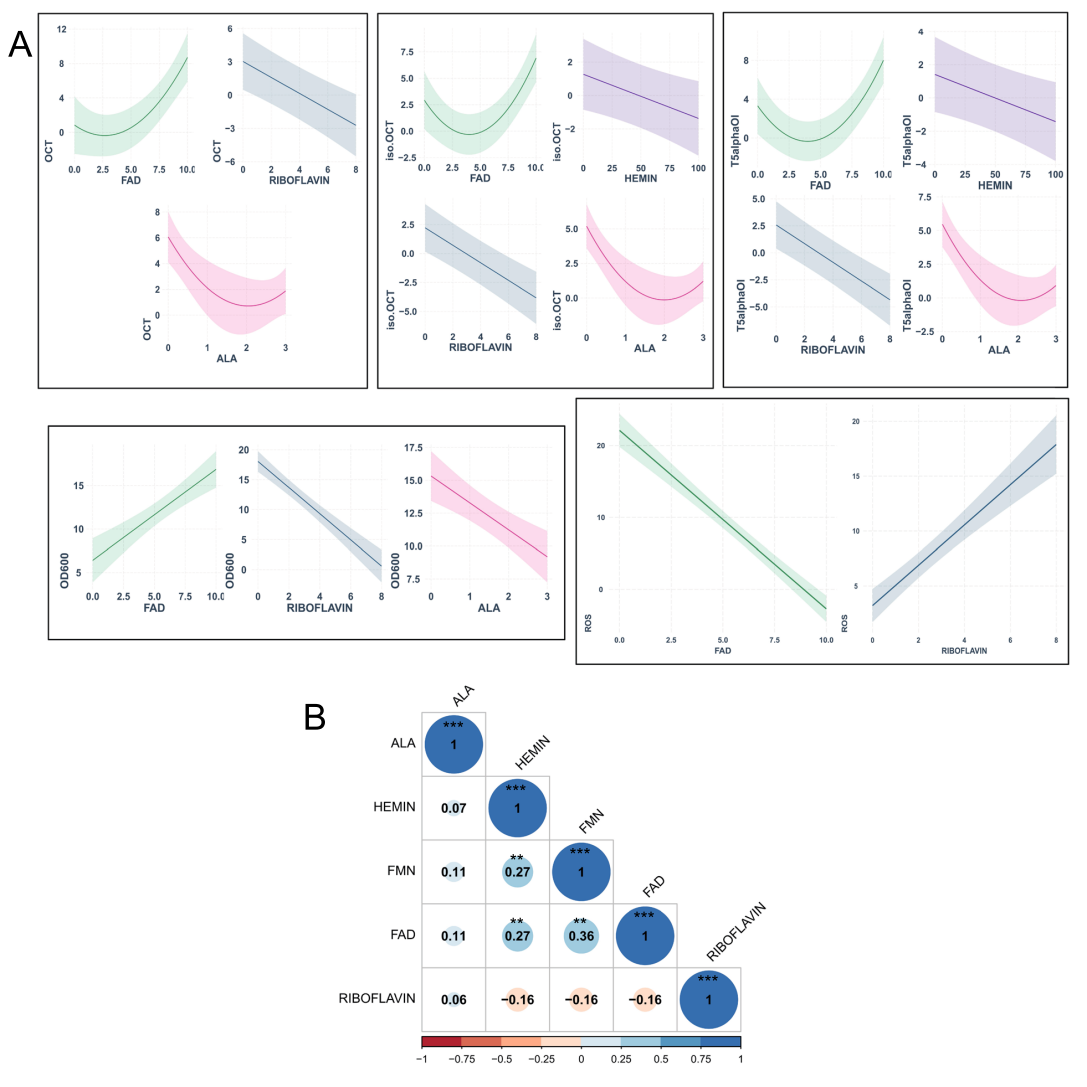
The plots of predicted models for oxygenated taxanes, biomass accumulation and ROS level in BN6 strain expressing *Taxus cuspidata CYP725A4* and reductase in tandem. **A** Effect plots showing the regression-based fitted values for each response relative to the concentrations of the predictors when supplemented in the growth medium (unit: mM for all predictors, except for hemin: µM); **B** Correlation plot for variables, showing the correlation coefficients between all the screened factors (navy blue and maroon represent the strongest positive and negative associations, respectively). The asterisks indicate significant differences in each pair of correlations (***p < 0.001, **p < 0.01, *p < 0.05). T5alphaOl and iso.OCT represent T5α-ol and iso-OCT. Figure was created with BioRender.com

Despite our incomplete understanding of the underlying mechanism for heme prosthetic group incorporation into the apocytochrome form of CYP725A4, it was critical to investigate if the flavins were the controlling elements for CYP725A4 optimal activity as a result of improved POR activity. In parallel, to screen for the potential effect of each parameter on the product distribution, a three-level definitive screening design [53] was performed in two-steps. The tested variables included the addition of ready heme (hemin) and iron precursor (δ-Aminolevulinic acid (ALA)) as well as flavin adenine dinucleotide (FAD), flavin mononucleotide (FMN) and their precursors, riboflavin. Cross-validation was then used to divide the data points into multiple subsets for training and validating the data and for improving the model accuracy. Then, the forward stepwise selection procedure was carried out to determine the predictors for each of OCT, iso-OCT, T5α-ol and biomass, where a 5% significance level was used to enter and eliminate each variable at first-order, followed by applying the same filtering procedure for the interaction terms. The prediction of the models and the raw data are included in Additional file 1: Figs. S6 and S7. While all these parameters are related in biological pathways, a correlation analysis depicted that although FMN, FAD and Hemin were weakly, but statistically significant correlated, in general, these variables could independently predict the response factor to strengthen our final conclusions (Fig. 5B). The linear model formulas for each response factor are listed in Additional file 1: Table S7 and the respective effect plots are illustrated in Fig. 5A.

For all the oxygenated taxanes, ALA was a significant negative predictor (OCT: p-value = 5.30e−05; iso-OCT: p = 2.2e−09; T5α-ol: p = 3.6e−10). However, hemin, which is the heme ferric chloride species (or ferrous protoporphyrin IX) [54], was found to significantly negatively influence the iso-OCT (p = 2.8e−06) and T5α-ol formation (p = 5.17e−10), as a separate factor, but it had a promoting effect in interaction with ALA (Fig. 5A). The negative impacts of iron factors were opposite to their remarkable effect on P450 activity reported in earlier works [54–57]. While yeast can regulate the heme biosynthesis effectively, the heme depletion reduces the total active P450 enzymes and increases the protein misfolding, leading to decreased growth rate [52, 56]. Additionally, under heme depletion, the cell expression profile resembles that of anaerobic growth, rendering it unfavourable to CYP725A4 monooxygenase, which is in addition to heme synthesis repressing impact of glucose and galactose during the fermentation [58]. While hemin is generally found to exert less positive effect on P450 product titre than ALA [38], no further improvement in product titres by this factor might be due to expression not being rate-limiting or the inactive portion of P450 enzyme being higher [52]. Alternatively, the supplemented iron might have been used by other iron-sulfur assembly systems in iron trafficking processes, like by iron-sulfur containing enzymes involved in DNA repair and replication to combat the oxidative stress [59]. It is argued that the hemin iron complex and ALA can cause lipid peroxidation [60] and ROS generation, respectively [61], resulting in metalloproteins inhibition [62], labile heme release [54], and P450 aggregation [63]. This is supported by the model predicted for biomass, which illustrated the negative effect of ALA on final biomass (Fig. 5A). However, our end-point measurement of ROS did not identify any of iron sources being significant predictors for ROS, rendering the cellular-level ROS decreasing the oxygenated taxanes production a weak phenomenon up to now or the iron factors were not involved in a change in the ROS level, at their selected concentrations.

Among flavins, FAD and riboflavin were found as significant factors (OCT: p-value = 2.4e−08, 1.1e−05; iso-OCT: p = 0.0001, 4.2e−09; T5α-ol: p = 2.6e−05, 5.2e−10) (Fig. 5A). While iron and flavin metabolisms are connected, their relative binding domains in the P450 and reductase proteins interact in electron transfer and oxygen activation [33]. It is known that P450s interact with POR through a combination of FMN-domain binding motifs in a specific isoform and FMN domain is a determining factor for P450-POR docking in endoplasmic reticulum (ER) membrane [64]. However, we did not find any significant contribution from FMN towards the oxygenated taxanes titres. Despite this, FAD was found influential in improving the final titre of all oxygenated taxanes, particularly at concentrations more than 5 mM, as suggested by the models’ terms coefficients (Additional file 1: Table S7). Interestingly, the interaction between FAD and riboflavin was found as a significant predictor (p = 5.0e−06) for ROS formation, which was in line with previous study by Chen et al. [65], where FAD was found to reduce the toxicity in *S. cerevisiae*. We noted that riboflavin exerted a negative impact on oxygenated taxanes production while it had a positive impact on increased ROS level, despite being known for its protecting effect for glutathione redox cycle improvement [65]. Due to the shared predictors, it is therefore possible that ROS is likely to have some impact on the overall diminished performance of yeast for oxygenated taxanes production. In line with this conclusion, the positive and negative impact of FAD and riboflavin on biomass accumulation, respectively, is also evident in the biomass model (FAD: p = 5.1e−07; Riboflavin: p = 3.4e−16).

**Fig. 6:**
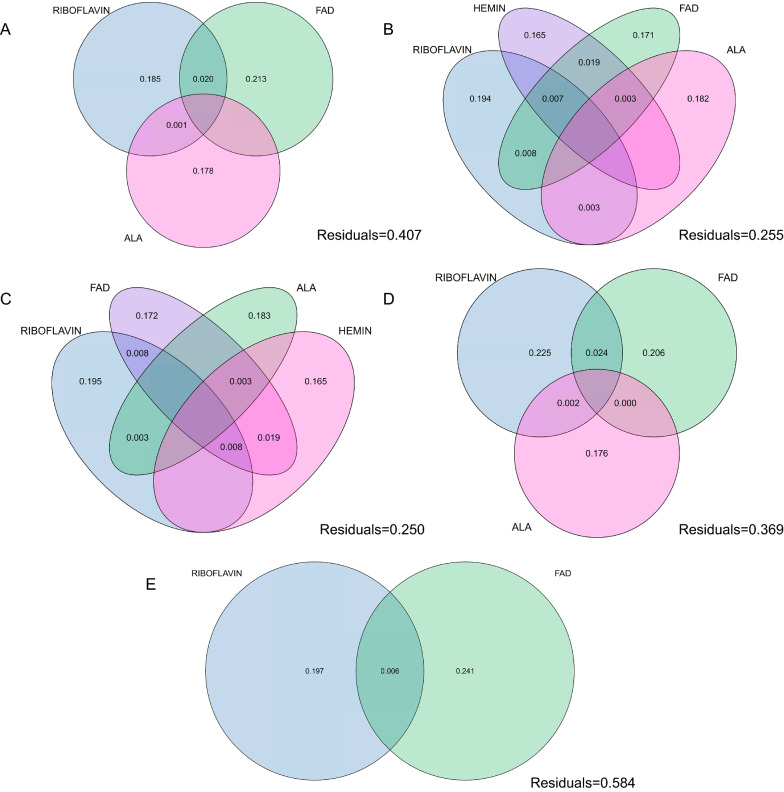
Venn diagrams of variation partitioning analysis of definitive screening design study for **A** OCT; **B** iso-OCT; **C** T5α-ol; **D** OD_600_ and **E** ROS. Venn graphs show the percentage of variation explained by each predictor for all response factors. The residual values (0 ≤ x ≤ 1, where x is the residual) are shown in the lower right corner. Figure was created with BioRender.com

To expand our previous models, it was next essential to understand the importance of each predictor on the overall response parameter (ALA: negative for all diterpenoids; hemin: negative for iso-OCT and T5α-ol; riboflavin: negative for all diterpenoids and biomass; FAD: positive for all diterpenoids and biomass). Hence, a variation partitioning study was performed to examine the contribution of each predictor to the overall variance in each response. As a result, each variable was found significant at p = 0.001 by the ANOVA test, regardless of the effect of other significant predictors. Hence, the importance of each predictor reported in each model was again validated (Fig. 6A–D). Similar to what the model predicted, ALA and hemin interaction were found to only explain a negligible percentage of total variance for both iso-OCT and T5α-ol (0.3%). In this study, we did not observe any strong correlation (OCT: − 0.28, p = 4.5e−05; iso-OCT: − 0.17, p = 0.01; T5α-ol: − 0.17, p = 0.01) between total cellular ROS level and oxygenated taxanes to explain the results, despite them sharing the same predictors of FAD and riboflavin in their regression models. Our variation partitioning model also illustrated that while only two variables could explain the ROS dataset, up to 58% of response variance could not be explained by the defined parameters of the study (Fig. 6E), highlighting the potential impact of other factors that could not be controlled in our study. Therefore, while we do not reject the impact of ROS on oxygenated taxanes production, evidently, further studies are required to quantify ROS by P450-POR (uncoupling) to untangle its contribution to diterpenoid formation more accurately. However, obviously, the molecular-level impact of cofactors on CYP725A4-POR performance is undeniable.

### Effect of increased salt and antioxidant concentrations on CYP725A4 activity

Based on the initial conclusions drawn from the previous sections, our fourth hypothesis was that the *Taxus* CYP725A4-POR enzymes both induce oxidative stress through uncoupling, whilst protecting against it [66] through mechanism-based inactivation that results in more reactive metabolites [67] from the substrates. These hypotheses were therefore tested by supplementing the BN6 strain growth medium with ascorbic acid antioxidant at different concentration levels, as it was shown to reduce the uncoupling-induced ROS production by cytochrome P450 enzyme [49, 68]. Similarly, as ionic strength has been shown to increase the uncoupling rate [69] and to potentially improve the P450 enzyme activity due to enhanced electron transfer [70], similarly, sodium chloride salt was also supplemented at different concentrations in BN6 growth medium (YPG) to study the changes in oxygenated taxanes production.

**Fig. 7:**
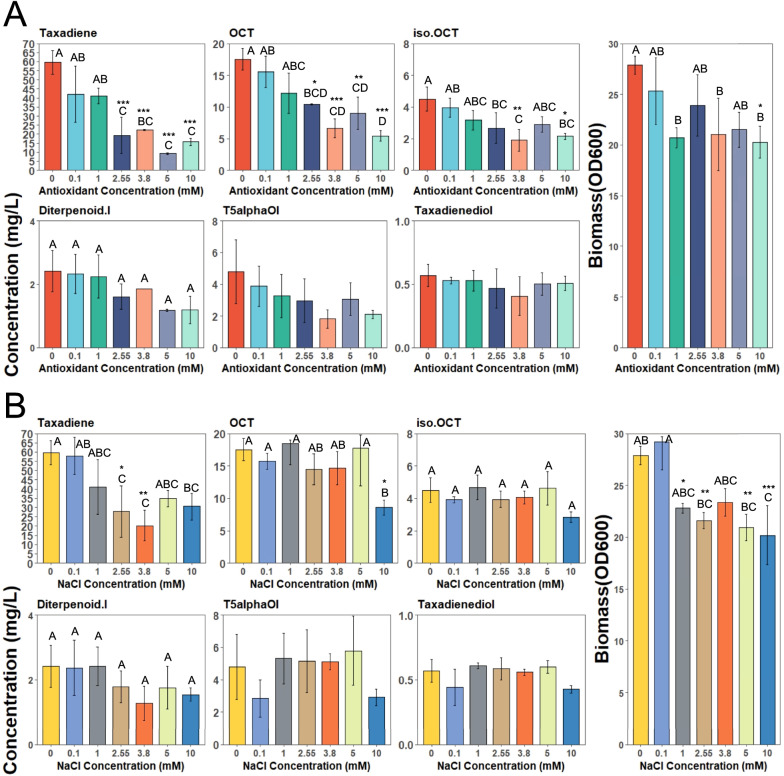
The effect of exogenous **A** antioxidant (ascorbic acid) and **B** NaCl salt addition on taxanes and biomass accumulation of BN6 strains, cultivated in YPG, by the end of 72-h growth in microplate. YPG is a rich medium and may have contained trace amounts of NaCl. The bars represent mean ± SD of triplicates. Tukey's HSD post hoc test shows a measure of difference across the whole strain groups, where different letters indicate significant differences in means (p < 0.05). The asterisks indicate significant differences relative to control condition without the antioxidant/NaCl supplementation (***p < 0.001, **p < 0.01, *p < 0.05 (adjusted p values)). T5alphaOl, iso.OCT and Diterpenoid I represent T5α-ol, iso-OCT and diterpenoid I. Figure was created with BioRender.com

A one-way ANOVA test revealed that except T5α-ol and taxadienediol, all other factors including OCT (p = 2.6e−05), iso-OCT (p = 0.003), taxadiene (p = 1.04e−05), diterpenoid I (p = 0.02) and biomass (p = 0.01) were significantly affected by the addition of ascorbic acid to the medium (Fig. 7A). The pairwise t-test with the control showed that OCT and taxadiene titres both significantly decreased upon increasing the concentration of ascorbic acid to 2.55 mM and higher (adjusted p < 0.05). Also, iso-OCT concentration was decreased upon adding 3.8 mM ascorbic acid in the medium (adjusted p < 0.05), except that at 5 mM, the iso-OCT concentration was not different to that of control. While we did not observe any significant drop in T5α-ol and taxadienediol titres, the biomass accumulation was not different at any level except when ascorbic acid reached final concentration of 10 mM (adjusted p = 0.03). As OCT was the most affected oxygenated product, it can be concluded that it might be the initial product of CYP725A4 enzyme as mentioned earlier. Since the biomass was not significantly affected by the ascorbic acid addition except at 10 mM concentration, the drop in taxadiene and oxygenated taxanes in other levels cannot be attributed to reduced biomass accumulation as stated earlier. Therefore, at least at 5 mM level, the 1.4-fold decrease in total oxygenated taxanes production (16.7 ± 4.2 mg/L) could be a result of potentially poor enzymatic performance and lower substrate conversion [49]. The presence of uncoupling events might support the activity of *Taxus* CYP725A4 and POR interaction in yeast as in Kells et al. [71] study, where the resulting reactive metabolites potentially act as the oxygen and electron donors to the P450 enzyme in addition to the molecular oxygen [47].

As illustrated in Fig. 7B, NaCl salt did not exert any significant impact on the final concentration of T5α-ol and taxadienediol. However, all other parameters including OCT (adjusted p = 0.001), iso-OCT (adjusted p = 0.013), taxadiene (adjusted p = 0.012), diterpenoid I (adjusted p = 0.03) and biomass (adjusted p = 3.8e−4) were all significantly affected by the addition of sodium chloride salt to the medium. A pairwise t-test comparison to the control level only depicted the significant decrease in OCT titre only at 10 mM NaCl (adjusted p = 0.01), and taxadiene concentration at 2.55 mM (adjusted p = 0.03) and 3.8 mM (adjusted p = 0.005) of NaCl in growth medium, without any significant impact at higher levels. Interestingly, except at 3.8 mM, in all other treatment groups, the biomass significantly decreased compared to control level (adjusted p < 0.05). While Tukey post hoc test revealed that the final biomass decreased as a result of increased ionic strength in as little as 1 mM, the overall oxygenated taxanes production per OD_600_ unit was found to increase by around 1.4-fold upon supplementing the exogenous NaCl concentration up to 5 mM, while a sudden drop to 0.81 ± 0.07 mg/L/OD_600_ was observed at 10 mM NaCl. The previous studies have reported on improved P450-POR interaction and intra-reductase electron transfer due to enhanced charge pairing and/or protein conformations, as a result of which the P450s performances were improved at increased ionic strengths [37, 38, 64, 72, 73]. Alternatively, the ionic strength might have improved the POR structural changes which favoured its electron transfer to CYP725A4 [69, 74, 75]. Therefore, the possibility of electrostatic forces also being determining in oxygenated taxanes production of CYP725A4 is likely as it was shown for *E. coli* cell-surface immobilised human CYP1A2 and POR [38]. Despite this, as proposed earlier, the uncoupling seems to play a minor effect on T5α-ol formation. However, further experiments are needed to provide a deeper insight on this point.

### Resting cell assay

Besides the potential uncoupling event by *Taxus* CYP725A4 and POR, the lipophilic nature of taxadiene and its derivatives might increase their intracellular residence, leading to cytotoxicity due to their metabolism, by which the endogenous mechanisms promote their functionalisation and conjugations to render them more polar [67]. Therefore, our fifth hypothesis was formulated based on the possibility that the endogenous pathways could contribute to overall CYP725A4 activity in yeast. In detail, it was hypothesised that some transient oxidised metabolites were formed and degraded quickly before being released into dodecane during yeast biphasic culture or the slow activity of P450 enzyme yielded different product profiles and limited the taxadiene conversion rate [14, 76]. In response to this hypothesis, we did not detect any leftover of the oxygenated taxanes in the cell pellets of the control group from the previous sections and only an insubstantial amount of taxadiene was found to be present (0.37 ± 0.05 mg/L). Since our study about medium type effect was conducted while the yeast was growing, we postulated that conducting the resting cell assays might be a better tool for improving our insights about CYP725A4 activity in *S. cerevisiae*.

Therefore, to illustrate all the metabolites of CYP725A4, BN6 resting cell assays in 50 mM phosphate buffer at approximate pH of 6–8 were performed to keep the effect of endogenous pathways on the enzyme activity to the minimum. Our initial results revealed that pH 6 was the least optimal for total oxygenated taxanes production (p = 0.04). Also, the yeast biomass increased after 115 h in pH = 8, while showing an almost double overall side oxygenated taxane: T5α-ol (22.5 ± 4) at time point one relative to pH = 7. However, the overall difference between pH = 7 and pH = 8 was insignificant (adjusted p = 0.69) (Additional file 1: Fig. S8). Also, it has been reported that the neutral pH is optimal for CYP725A4 activity, where this enzyme shows half maximal activity at more acid or basic environments [77]. Despite this, the optimal acidity for *Taxus* POR has not been reported yet.

**Fig. 8:**
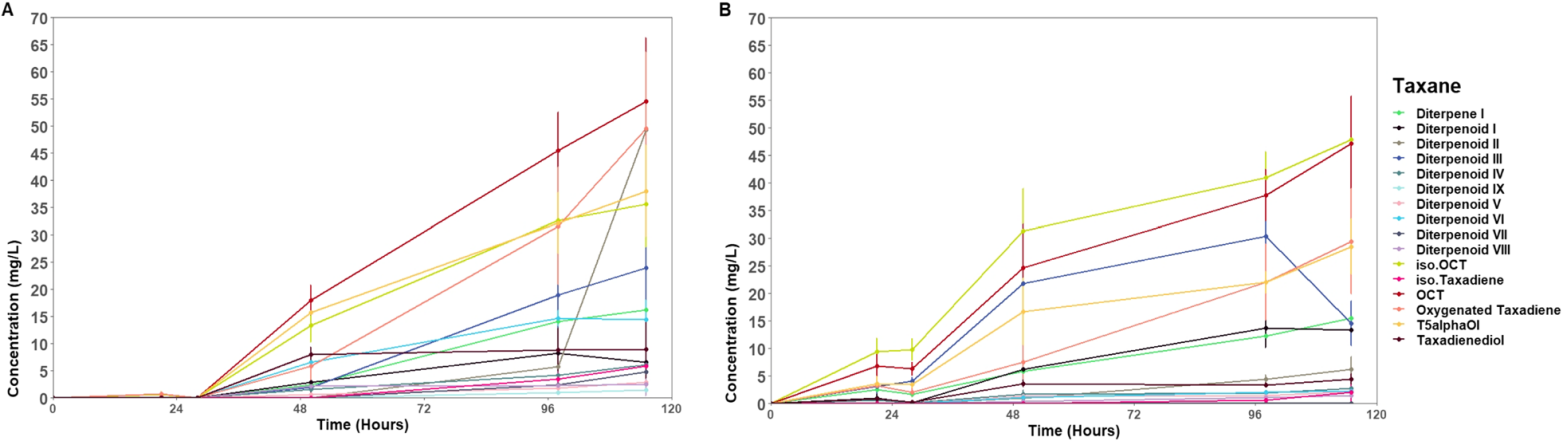
Resting cell assay of BN6 strain expressing *Taxus cuspidata CYP725A4* and *POR*. **A** The concentration of terpenes and terpenoids by galactose induction **A** at the resting stage (post-rest induction) and **B** during the growth stage (pre-rest induction). The kinetic changes of relevant taxanes represent mean ± SD of triplicates over a five-day run of 115 h. The assays were both performed using 10 mL of 50 mM potassium phosphate buffer (pH = 7), containing 20% (v/v) dodecane. The starting biomass (OD_600_) was 40 and the biomass accumulation was not changed by the end of 115 h. Iso.Taxadiene*,* t5alphaOl and iso.OCT represent iso-taxadiene*,* T5α-ol and iso-OCT. Figure was created with BioRender.com

The subsequent experiments were then performed using 50 mM phosphate buffer at pH = 7. Two galactose induction schemes were then designed. In the first one, which we called pre-rest, the galactose was used for both growth and taxanes production and this carbon source was therefore excluded from the buffer during the resting stage. For the other one, which we called post-rest, glucose was used to only grow the taxane-producing BN6 strain to obtain sufficient biomass. Then, the buffer was supplemented with galactose (2% (w/v)) to induce the taxanes production during the resting stage. As the biomass accumulation did not change over time at pH 7, it can be inferred that the higher NADPH availability together with optimal pH favoured higher and more diverse oxygenated taxanes production, where a relatively higher total oxygenated taxanes titre at the last end point (115 h) of pre-rest galactose induction (354.6 ± 51.1 mg/L, 8.9 ± 1.3 mg/L/OD_600_) (Fig. 8B) was achieved, compared to that of post-rest galactose induction (220.5 ± 34.2, 5.51 ± 0.9 mg/L/OD_600_) (Fig. 8A). This could be due to suboptimal performance of metabolite trafficking and galactose metabolism in the inactive cells from the post-rest galactose induction set. Also, the titre of all taxanes, including taxadiene, increased over 115 h in both resting cell assays. Interestingly, by the end of the experiment, the major products of the post-rest induction were iso-OCT (50.4 ± 5.9 mg/L), OCT (43.5 ± 5.4 mg/L) and T5α-ol (30.2 ± 4.3 mg/L) (Fig. 8A). However, the major products of the pre-rest induction were in order of OCT (54.6 ± 11.7), T5α-ol (38.1 ± 8.4) and iso-OCT (35.7 ± 7.8) (Fig. 8B). Therefore, OCT remained as the major product, while both iso-OCT and T5α-ol were the late products of the CYP725A4 enzyme (Additional file 1: Fig. S9). Surprisingly, having a peak of 288 (m/z), which corresponds to the molecular weight of monooxygenated taxanes [7], taxadiene was also found to be hydroxylated. Hence, it is called oxygenated taxadiene in this study. This denotes that the availability of cofactors and optimal resources do not necessarily result in the improved production of major CYP725A4 product. Since we did not observe any change in the yeast biomass at the endpoint, the increase in taxadiene could be justified by enzyme folding events, or the P450 uncoupling, which renders the substrate presence a less important factor for the enzymatic catalysis [63], or the mechanism-based inactivation of the taxadiene into more reactive metabolites by CYP725A4 which eventually hinders the proper activity of the CYP725A4 enzyme. However, the later seems unlikely as the P450-POR were still active until the experiment end point. Aside from these points, the upstream pathways producing the taxadiene precursor, geranylgeranyl diphosphate (GGPP), might have also become more active during the resting stage and increased taxadiene production flux. Hence, although the titres of oxygenated taxanes and taxadiene isomers had increasing trends over 115 h, the low substrate conversion did not seem to be solely the result of the slow CYP725A4 activity.

Despite this, it is possible that the OCT and iso-OCT are the intermediates to the downstream pathways in *Taxus* sp., or they are more toxic than T5α-ol, being degraded by the plant underground metabolism. This hypothesis becomes stronger as Edgar et al. [15] showed that only a minor amount of OCT was observed in *Taxus* plant cell culture. Therefore, it could be postulated that all these three oxygenated taxanes are the major products of CYP725A4, while the other diterpenoids with lower quantities might have been formed as a result of oxidation, which has a higher energy barrier in comparison to the epoxidation process [67]. As such, some of these oxygenated compounds could be transient metabolites (metabolons) that degrade into more stable compounds [15], which were not metabolised at the resting stage or are the late products due to higher NADPH and cofactor availability for taxanes production, or are formed due to structural rearrangements [78]. As we found at least ten additional other oxygenated taxanes in both resting assays with 288 (m/z) (Additional file 1: Figs. S10–S12), while having only five time points, we conducted a Granger causality test to find the correlation and causality relationships among them including taxadiene isomer(s) [79]. To ensure the results were not impacted by the yeast growth, only post-rest results were subjected to Granger test. According to the test results, there was a significant two-way association between OCT and iso-OCT (p = 0. 04 and 0.04). This was in line with a previous report, where iso-OCT was reported to be potentially formed by the structural rearrangement of OCT [80].

Focusing on the late oxygenated taxanes, labelled with roman numbers due to their novelty, iso-taxadiene was found as Granger-causal of diterpenoid III (p = 0.04), and oxygenated taxadiene and potentially taxadiene that might have co-eluted with it, was found as the Granger-causal of diterpenoid IX (p = 0.02) and diterpenoid VII (p = 0.04). Interestingly, diterpenoid IX was also found to be associated with diterpenoid III (p = 0.045), and taxadienediol, which is a double oxygenated taxane (304 (m/z)), was Granger-caused by diterpenoid VII (p = 0.03). While the complete identity of this taxadienediol is not unknown, Edgar et al. [81] have proposed two different taxadienediols, the first one being taxadien-5α-10β-diol which is one of the primary dihydroxylation products of CYP725A4 enzyme and another being 5(12)-oxa-3(11)-cyclo-taxan-10-ol, which was justified to be formed upon epoxide hydroxylation as a result of change in the enzyme selectivity. Hence, it is possible that any of the new dioxygenated diterpenoids be either the hydroxylated product of an oxidised or epoxidised intermediate.

Interestingly, it was also found that diterpenoid III unidirectionally Granger-caused iso-taxadiene (p = 0.03) and similarly, diterpenoid VII and IX Granger-caused the oxygenated taxadiene (p = 0.03 and 0.05, respectively). Therefore, it is also possible that these diterpenoids stimulate the higher production of taxadiene isomers. Overall, the concentration of total taxanes during post-rest stage (361.4 ± 52.4 mg/L) in a 10-mL scale was remarkable and comparable to the previous study, reporting the highest total taxanes concentration by whole *S. cerevisiae* culture at 1-L scale with optimised cultivation medium [13].

## Conclusions

CYP725A4 is the first committed cytochrome P450 enzyme in Taxol® (paclitaxel) biosynthetic pathway. It catalyses the conversion of taxadiene into taxadiene-5α-ol (T5α-ol), as the main product, and several other diterpenoids, mainly 5(12)-oxa-3(11)-cyclotaxane (OCT), 5(11)-oxa-3(11)-cyclotaxane (iso-OCT) and taxadienediols. There are several reasons for which this enzyme has been known as a bottleneck to the whole Taxol® pathway integration in non-native systems, mainly due to its slow activity and the high side-product: main-product titre ratio. While this enzyme was long known to undergo radical-rebound mechanism, the recent studies have proposed the existence of an alternative catalysis route, namely the epoxidation. We therefore started by examining the effect of *Taxus cuspidata CYP725A4* and reductase (*POR*) copy numbers in *S. cerevisiae*. While using low-glucose induced promoter, pHXT7, for *CYP725A4* expression almost doubled the total oxygenated products to 17.8 ± 1.3 mg/L in a taxadiene-producing yeast strain, a similar result was achieved (18.6 ± 5.5 mg/L) by expressing an extra copy of *CYP725A4* under the pGAL1 promoter in a yeast also expressing the next Taxol® pathway gene, Taxa-4(20),11(12)-dien-5α-olO-Acetyl Transferase (*TAT*). This however did not affect the product distribution and the T5α-ol titre compared to the control strain. Interestingly, we found that *TAT* expression likely promoted the consumption of all major CYP725A4 diterpenoids, where further investigation would however be required. In contrast, an extra copy of *POR* resulted in an unstable yeast strain, highlighting the inefficiency of gene dosage variance in improving the enzyme productivity, and higher main product titre. By assuming that the epoxidation route was directly correlated to the cytochrome P450 uncoupling event, we compared the performance of the strains harbouring the tandem and fusion gene cassettes of *CYP725A4* and *T. cuspidata POR* to those expressing the fusion gene cassettes of *CYP725A4* and self-sufficient prokaryotic reductases with known strong electron coupling capacity. Interestingly, the separate expression of *Taxus CYP725A4* and *POR* outperformed the fusion constructs in terms of whole oxygenated taxanes concentrations, being 6.1 ± 1.4 mg/L compared to 56.8 ± 17.6 mg/L, favouring the hypothesis of uncoupling being influential for the formation of whole diterpenoids. Despite this, OCT was the main product in all oxygenated taxanes-producing strains, while a shift towards higher T5α-ol formation and taxadiene consumption was noticed using *T. cuspidata* CYP725A4 and POR fusion. Using a variety of other growth media, despite affecting the final biomass, did not result in consensus changes in the ratio of T5α-ol: side oxygenated taxanes, and OCT remained the major product. Thus, the previous attempts to increase the biomass as a proxy for increased diterpenoid titres are questioned. To screen for the rate-limiting step in the strain expressing the *Taxus CYP725A4* and *POR* in tandem, five cofactors involved in the structural components of their enzymes were tested. These included hemin (ready heme), iron precursor (δ-Aminolevulinic acid), flavin adenine dinucleotide (FAD), flavin mononucleotide (FMN) and their precursor, riboflavin, all screened by an augmented definitive screening design. Forward stepwise regression modelling revealed the positive impact of FAD in oxygenated taxanes and biomass improvement, while riboflavin and ALA were found to have negative impacts on the same responses. Also, hemin was found to further decrease the concentrations of iso-OCT and T5α-ol. However, due to positive effect of ALA and hemin, we anticipate that the two former oxygenated products were the secondary metabolites of CYP725A4. Studying the effect of antioxidant and ionic strength on P450-POR activity further strengthened the uncoupling playing the main factor in oxygenated taxanes production, releasing electron and oxygen sources for use by the enzyme. Finally, the resting cell assays were employed to study the reaction kinetics with minimised effect from the yeast cell internal metabolic processes in a strain expressing the *CYP725A4* and *POR* in tandem. This led to the highest taxanes concentrations in *S. cerevisiae* (361.4 ± 52.4 mg/L) at 10-mL scale using galactose inducer before the resting stage. As a consequence of reduced activities of the growth-associated processes and other cellular mechanisms, around 4.4-fold difference in total taxanes concentration was observed in comparison to the growing cells in YPG medium. In addition, a diverse variety of products which were not detected by whole yeast culture, was revealed, rendering the resting cell assay a valuable tool for studying the activity of Taxol® downstream enzymes in yeast. Moreover, as the iron, flavins, antioxidants and salt were found to impact both CYP725A4 and the whole cell, future studies should invest on finding ways to assure sufficient reducing equivalents and cofactors are provided through more cost-effective approaches like genetic engineering for more efficient electron transfer and hydroxylation. In conclusion, based on our results and in particular due to the strong harmful effect of *CYP725A4* and *POR* overexpression on the yeast, future studies need to construct more efficient fusion constructs to tightly regulate the oxygenated taxanes production with respect to uncoupling effect, whilst maximising the taxadiene consumption.

## Methods

### Yeast strains

The parent *S. cerevisiae* strain used for genomic integration of CYP725A4 and reductases was LRS5 (MATa, *leu2-3, 112::HIS3MX6-pGAL1-ERG19/pGAL10-ERG8; ura3-52::URA3-pGAL1-MvaSA110G/pGAL10-MvaE* (codon-optimised); *his3Δ1::hphMX4-pGAL1-ERG12/pGAL10-IDI1;trp1-289::TRP1_pGAL1-CrtE(Xanthophyllomyces dendrorhous)/pGAL10-ERG20; YPRCdelta15::NatMX-pGAL1-CrtE(*codon-optimised)/pGAL10-CrtE:: ARS1014a::pGal1-TASY-GFP:: ARS1622:: pGal1-MBP-TASY-ERG20*::ARS1114a::pGal1-MBP-TASY-ERG20*) previously constructed in [12]*,* originated from CEN.PK2-1C (EUROSCARF collection). To study the gene dosage effect, LRS6 (LRS5 *ARS511b::pGAL1-CYP725A4-PGK1t/pGAL3-POR-ENO2t; RKC3::pGAL1-TAT-CYC1t*) was used [14]. The constructed strains are listed in Table 1 and schematic representations are provided in Additional file 1: Table S1.

**Table 1:** The details and schematic representations of the used strains in this study

Strain name	Genotype	References
LRS5	MATa, leu2-3,112::HIS3MX6-pGAL1-ERG19/pGAL10-ERG8; ura3-52::URA3-pGAL1-MvaSA110G/pGAL10-MvaE (codon-optimised); his3Δ1::hphMX4-pGAL1-ERG12/pGAL10-IDI1;trp1-289::TRP1_pGAL1-CrtE(X. dendrorhous)/pGAL10-ERG20; YPRCdelta15::NatMX-pGAL1-CrtE(codon-optimised)/pGAL10-CrtE:: ARS1014a::pGAL1-TASY-GFP:: ARS1622:: pGAL1-MBP-TASY-ERG20*::ARS1114a::pGAL1-MBP-TASY-ERG20*	[12]
LRS6	LRS5 *ARS511b::pGAL1-CYP725A4-PGK1t/pGAL3-POR-ENO2t; RKC3::pGAL1-TAT-CYC1t*	[14]
BN1	LRS6 *ARS416d::pGAL1-CYP725A4-PGK1t*	This study
BN2	LRS6 *ARS416d::pGal3-POR-ENO2t*	This study
BN3	LRS5 *ARS511b::pHXT7-CYP725A4-PGK1t/pGAL3-POR-ENO2t*	This study
BN4	LRS6 *ARS416d::pTRX2-CYP725A4-PGK1t/pGAL3-POR-ENO2t*	This study
BN5	LRS5 *X-2::pGAL1-CP725A4-RPL41Bt*	This study
BN6	LRS5 *X-2::pGAL1-CP725A4-RPL41Bt/pTDH3-POR-ICY2t*	This study
BNF-1	LRS5 *X-2::pGAL1-CP725A4-L1-POR-RPL41Bt*	This study
BNF-2	LRS5 *X-2::pGAL1-CP725A4-L2-EcfldA-L3-EcFpr-RPL41Bt*	This study
BNF-3	LRS5 *X-2::pGAL1-CP725A4-L4-AxRED*(codon-optimised)-*RPL41Bt*	This study
BNF-4	LRS5 *X-2::pGAL1-CP725A4-L5-L6-RhFRED*(codon-optimised)-*RPL41Bt*	This study
BNF-5	LRS5 *X-2::pGAL1-CP725A4-L7-BMR*(codon-optimised)-*RPL41Bt*	This study
BNF-6	LRS5 *X-2::pGAL1-CP725A4-AxRED*(codon-optimised)-*RPL41Bt*	This study
BNF-7	LRS5 *X-2::pGAL1-CP725A4-POR-RPL41Bt*	This study
BNF-8	LRS5 *X-2::pGAL1-CP725A4-truncated POR-RPL41Bt*	This study

The ″L″ represents the linker as listed in Additional file 1: Table S5

### Growth media and cultivation platforms

The synthetic defined medium consisted of 0.79 g/L Complete Supplement Mixture (CSM) powder (Formedium), 5 g/L ammonium sulphate (Fisher Chemical™), 1.7 g/L yeast nitrogen base without amino acids (Alfa Aesar™) and was supplemented with 2% (w/v) glucose (SDD for resting cell assay; Thermo Scientific™) or galactose (SDG for resting cell assay and definitive screening; Thermo Scientific™). The dropout medium for yeast transformation had the same formula for SDD, except that it contained 0.77 g/L CSM-URA (MP Biomedicals) and 20 g/L agar (ACROS Organics™). The routinely used growth medium containing yeast extract (1%(w/v)) and casein peptone (2%(w/v)) (Merck), supplemented with 2% (w/v) glucose (YPD) or galactose (YPG) was used for inoculum preparation and production screening, respectively. Two other media, Brain Heart Infusion Broth-galactose (BHIG, Merck) and Terrific Broth, Modified-galactose (TBMG, Merck) were included to study the medium effect. BHIG contained 5 g/L beef heart (infusion from 250 g), 12.5 g/L calf brains (infusion from 200 g), 2.5 g/L Na_2_HPO_4_, 2 g/L D( +)-glucose, 10 g/L peptone and 5 g/L sodium chloride. TBMG contained 12 g/L tryptone (pancreatic digest of casein), 24 g/L yeast extract, 9.4 g/L K_2_HPO4, 10 g/L glycerol, and 2.2 g/L KH_2_PO4. LBG contained 5 g/L NaCl, 10 g/L tryptone and 5 g/L yeast extract. Each medium was supplemented with 20 g/L galactose. A 20% (v/v) *n*-dodecane (ACROS Organics™) was used in all cultivations to extract taxanes and prevent their air-stripping. Gene dosage study was initially performed in 24-well deep well polypropylene plates. However, due to incompatibility of polypropylene and dodecane and massive co-elution of taxane products and contaminant peaks (Additional file 1: Fig. S1), the results were deemed invalid. Therefore, all gene dosage study strains were screened in 50 mL YPG in 500 mL shake flasks covered with cotton wool and foil, and the primary screening for the next strains were performed in total of 5 mL including 20% (v/v) dodecane in 10 mL glass tubes, covered with cotton wool and foil.

### Yeast and bacteria strain construction

The native promoter and terminator sequences of yeast genome were used in this study. All primers and single stranded linker sequences were synthesised by IDT (Integrated DNA Technologies, Inc.). *S. cerevisiae* codon-optimised *Taxus cuspidata CYP725A4* and *POR* were amplified from LRS6 strain [14]. *Bacillus megaterium* P450_BM3_ Reductase (*BMR*), *Rhodococcus* sp. P450_RhF_ Reductase (*RhFRED*) and *Albidovulum xiamenense* CYP116B64 Reductase (*AxRED*), were codon optimised for *S. cerevisiae* using IDT Codon Optimisation Tool and all were then synthesised with natural linkers by TWIST Bioscience. *Escherichia coli* Flavodoxin (*fldA*) and Flavodoxin Reductase (*Fpr*) were directly amplified from DH5α cells. The truncated *POR* sequence was constructed by removing the transmembrane domain as predicted by TMHMM-2.0 [82]. To amplify DNAs, touchdown PCRs [83] were performed with Phusion Flash High-Fidelity PCR Master Mix (Thermo Scientific™). The GC-rich linear plasmids from the first CRISPR/Cas9 methodology [10] were amplified using the high-fidelity PrimeStar GXL DNA Polymerase (Takara Bio) for gene dosage study. In the second methodology, the single-stranded sgRNAs were first phosphorylated by T4 PNK (NEB) and T4 DNA ligase buffer (NEB), followed by annealing and ligation into linearised pWS082 vector according to Golden Gate methodology [84] and transformed into 10-beta competent *E. coli* (NEB) according to manufacturer’s instructions. Upon initial screening of transformants on LB Lennox (Merck) medium supplemented with ampicillin (Fisher BioReagents™) for GFP reporter exclusion, the plasmids of a few selected transformants were isolated with GeneJet Miniprep kit (Thermo Scientific™) and sequenced. Together with pWS158 Cas9 vector, they were then either amplified by a 3-step PCR reaction using Phusion Flash High-Fidelity PCR Master Mix (Thermo Scientific™) or 1 µg of each were digested with EcoRV (Promega) and BsmBI (NEB) for 3–4 h, respectively, to yield homology arms for repairing into single plasmid. The correct sizes of digested plasmids were recovered from 0.7% (w/v) low-melting point TopVision agarose (Thermo Scientific™) using Zymoclean Gel DNA Recovery Kit (Zymo Research). All PCR products and donor DNAs were purified with GeneJET PCR Purification Kit (Thermo Scientific™).

Self-sufficient CYP725A4 were constructed by phosphorylating and annealing the single-stranded linker sequences, using the same procedure described above. Following PCR amplification, this short fragment was purified with ethanol according to Sambrook and Russell [85]. The full gene cassettes containing pGAL1, CYP725A4 (without stop codon), linker (optional), (with start codon) reductase and RPL41Bt in pUC19 vector (NEB) were built by Gibson assembly using NEBuilder® HiFi DNA Assembly Cloning Kit (NEB), according to manufacturer’s guidelines. Upon transformation into competent DH5α cells (NEB) and screening on LB-Ampicillin plates, bacterial colony PCR was performed using Q5® High-Fidelity 2X Master Mix (NEB) according to manufacturer’s instructions to verify the correct assembly. The gene cassettes were then amplified with Phusion Flash High-Fidelity PCR Master Mix (Thermo Scientific™), flanked by homologous arms for integration locus X2 [86] and were sequenced (GENEWIZ Inc.) before the yeast transformation. The sequences of primers, genes, sgRNAs and linkers are listed in Additional file 1: Tables S2, S3, S4 and S5, respectively.

### Yeast transformation and strain construction

The strains with extra P450 and/or reductase copy numbers were constructed using Cas9-sgRNA 2-micron plasmids harbouring a *URA3* selection marker and for which single stranded sgRNAs were synthesized [10]. In this method, 200 ng of sgRNA, 100 ng of CRISPR plasmid and > 1000 ng of total linear DNA, including up and down sequences of integration loci, were used in equimolar amounts for each transformation according to standard lithium acetate protocol [87]. Yeast fusion strains and tandem expression strain were created using pWS082 (sgRNA entry vector) and pWS158 (Cas9 gap repair vector—URA3) plasmids. These plasmids were gifts from Tom Ellis (Addgene plasmid #90517; http://n2t.net/addgene:90517; RRID:Addgene_90517 and Addgene plasmid #90516; http://n2t.net/addgene:90516; RRID:Addgene_90516). To increase the transformation efficiency, a modified protocol by Muller et al. [88] was implemented for yeast transformation, where the harvested cell pellets were resuspended in sterile water and the heat shock lasted for 30 min at 42 °C. The concentrations of linear DNA fragments for homologous recombination were maintained at fragment size (bp)/2 [10] for each transformation and 1:2.5–3 linearized Cas9:sgRNA plasmids were utilized. Chromosomal integration at the target sites were confirmed by colony PCR using DreamTaq Green PCR Master Mix (2X) (Thermo Scientific™). Plasmid curing was subsequently performed on successful colonies through sequential culture in YPD liquid medium until no growth was observed on concurrent CSM-URA agar plates. Total genomic DNAs were then prepared using Yeast DNA Extraction Kit (Thermo Scientific™), followed by Sanger sequencing (GENEWIZ Inc.; Edinburgh Genomics).

### Effect of growth medium on CYP725A4 product profile

The BN6 cells were grown overnight in 5 mL of YPD medium, and with a starting OD_600_ = 1, they were then inoculated into different media, namely SDG, YPG, LBG, BHIG and TBMG (as described above), all containing dodecane at final 20% (v/v) concentration. The prepared tubes were covered with cotton wool and foil, and were incubated at 30 °C, 250 rpm, for up to 72 h prior to GC–MS analysis.

### Design of experiment: Definitive screening design (DSD)

A three-level definitive screening design [53] was created by Minitab software to study the effect of five cofactors on CYP725A4 and POR product profile and final biomass. Total 36 runs, split into two sets, each with controls, were performed in triplicates using the basal SDG medium to augment the model prediction power. The details of DSD runs are tabulated in Additional file 1: Table S6. The following factors were categorised into iron and flavin groups, and were supplemented in the final concentrations listed below:

First Run:

A) Iron: (i) Hemin: 10, 55, 100 µM; (ii) δ-Aminolevulinic acid (ALA): 0.1, 1.55, 3 mM.

B) Flavin: (i) Riboflavin: 0.1, 1.05, 2 mM; (ii) Flavin adenine dinucleotide (FAD): 5, 7.5, 10 mM; (iii) Flavin mononucleotide (FMN): 5, 7.5, 10 mM.

Second Run:

A) Iron: (i) Hemin: 5, 27.5, 50 µM; (ii) ALA: 0.1, 1.55, 3 mM.

B) Flavin: (i) Riboflavin: 1, 4.5, 8 mM; (ii) FAD: 1, 4.5, 8 mM; (iii) FMN: 1, 4.5, 8 mM.

All chemicals were sourced from Thermo Fisher Scientific (Thermo Scientific™) at the highest available purity. The starting cultures were prepared from overnight cultivation of BN6 strain in YPD. To ensure sufficient aeration and proper mixing, the total culture volumes were set to 560 µL, inoculated at OD_600_ = 1 and incubated at 30 °C and 1400 rpm for 72 h. A 140 µL of dodecane was also added at 20% (v/v) to reach 700 µL total volume. Each condition was tested in triplicate in a 96-well microplate with glass inserts (Hirschmann™), sealed with two layers of gas permeable adhesive plate seals (Thermo Scientific™), to minimise the dodecane evaporation. The results were analysed with GC–MS and the second run data were normalised relative to the control sets.

At the end of the cultivation, the total reactive oxygen species (ROS) was measured using Deep Red fluorometric intracellular ROS kit (Merck) according to manufacturer’s instructions in BMG Clariostar microplate reader. The relative fluorescence units were normalised by the final biomass.

### Statistical modelling of DSD study

The entire data was subjected to forward stepwise multiple regression modelling [53] using *lmStepAIC* function in caret package in R [89]. Full quadratic linear models with interaction terms for each of OCT, iso-OCT, T5α-ol, biomass and ROS were constructed and parametrised using cross-validation (k = 3 to 5, repeated 10 times) for automatic training and testing of the data. The model selection was performed and evaluated with the following criteria: 1) Minimum Akaike information criterion (AiCc); 2) Exclusion of variables with significant p-value of 0.05 or greater; 3) Controlling for multicollinearity of predictors [90] and selecting terms with variance inflation factor (VIF) below 5, using *VIF* function in car R package [91]; 4) Lowest model error like mean absolute error (MAE) and root mean square error (RMSE), and maximum explained variance by adjusted R square; 5) Model diagnostics was perfomed with *check_model* and *performance_accuracy* functions in performance R package [92] to check for homogeneity of variance, influential outliers, model fit and linearity, and prediction accuracy, respectively. To maintain the linearity assumption for ROS model, the outliers were removed prior to model construction. The constructed models with scaled data were then analysed for partition of variation (redundancy analysis) using *rda* function in vegan R package [93]. The correlations between variables were determined by *cor.test* function in R to calculate the Kendall’s tau correlation coefficient. The correlation plot and significance test were then visualised using corrplot R package [94].

### Effect of increased salt and antioxidant concentrations on CYP725A4 activity

The BN6 cells were grown overnight in 5 mL of YPD medium, and inoculated into YPG media, containing different concentration of either sodium chloride or ascorbic acid at regular intervals of 0.1, 1, 2.55, 3.8, 5 and 10 mM at a total volume of 700 uL including 20% dodecane. Triplicates of each run were prepared in a 96-well microplate with glass inserts (Hirschmann™), sealed with two layers of gas permeable adhesive plate seals (Thermo Scientific™) and incubated for 72 h at 30 °C and 1400 rpm.

### Resting cell assay

The BN6 cells were grown overnight in 500 mL of SDD or SDG and harvested by sequential centrifugation of every 50 mL at 5000 rpm for 2 min. The cell pellets were washed twice with sterile ultrapure water. The cell pellets at 40 OD_600_ units were then resuspended in 10 mL of 50 mM filter sterilised potassium phosphate buffer (pH = 7), containing 20% (v/v) dodecane, in glass tubes covered with cotton wool and aluminium foil. The cells were incubated at 30 °C, 250 rpm, and 50 µL of dodecane samples were withdrawn after 21, 28, 50, 98 and 115 h during this experiment for all three replicates. A parallel run with LRS5 was done in SDD to confirm and relate the observed peaks to *CYP725A4* and *POR* expressions. Prior to this experiment, preliminary runs of BN6 experiment were done to determine a suitable pH for CYP725A4 activity, following the same procedure except that it was done with pH = 6, 7, and 8 with galactose induction during the resting stage and with lower oxygen availability. The possible relationship among the taxanes over time were investigated using Granger causality testing [79] by lmtest R package [95]. For this, the data were scaled and significant data were selected at 95% confidence interval.

### Diterpenoid identification and quantification

At the end of each cultivation, 50 µL of dodecane overlay was extracted from the culture media and analysed by GC–MS using Trace 1300 GC (ThermoFisher Scientific), equipped with TG-SQC column (15 m × 0.25 mm × 0.25 μm) (gene dosage study) or TraceGOLD TG-5MS (30 m × 0.25 mm × 0.25 μm) (all other experiments). The mass spectra in the range of 40–650 m/z was recorded on a Thermo Scientific ISQ Series single quadrupole mass spectrometer using EI ionization mode. The GC temperature programme began at 120 °C (3 min) and was then raised to 250 °C at a rate of 20 °C/minute with 3-min hold time. Xcalibur™ software (ThermoFisher Scientific) was employed for data processing and AMDIS software was used for extracted ion (288 m/z) chromatograms retrieval. Geranylgeraniol (Sigma Aldrich; GGOH) was used to quantify GGOH concentration. Pure taxadiene (kindly supplied by Baran Lab, The Scripps Research Institute) and the diterpene totarol (Cayman Chemical) were used as standards for quantifying taxanes using shorter and longer GC columns, respectively.

### Quantification of intracellular oxygenated taxanes

The control cells used in antioxidant-ionic strength study were pelleted, vortexed and mixed with 1 mL of 99% acetone (Thermo Scientific™)-dodecane (1:1) for 16 h at 800 rpm. The extracts were centrifuged at 215 × g for 5 min, and the dodecane layer was extracted for GC–MS analysis.

### Statistical analyses

All statistical analyses of this study were performed in R version 4.1.3. Apart from the gene dosage study which was evaluated with two-way analysis of variance (ANOVA) to study the effect of both strain type and growth time, a one-way ANOVA was conducted to evaluate the other results with R stats package. Using Levene’s test in car R package [91, 96], the non-normal distributions at p-value < 0.05, were subjected to the nonparametric Kruskal–Wallis test with R stats package. All analyses were completed with Tukey’s HSD test in agricolae R package [97] for post-hoc comparisons after ANOVA analysis. Alternatively, the post hoc Dunn test in FSA R package [98] was done after Kruskal–Wallis test. Where a comparison to the control level was required, Bonferroni corrected pairwise-t-tests were carried out at significance of adjusted p < 0.05.

## Supplementary Information

**Additional file 1: Table S1.** Schematic representation of strains in this study. **Table S2.** List of primers used in the study. **Table S3.** Sequence of genes in this study. **Table S4.** List of guide RNA sequences used in this study. **Table S5.** List of linkers used in this study. **Table S6.** Augmented definitive screening designs for optimising P450-reductase expressions, including controls. **Table S7.** Linear model formulas for response factors in definitive screening designs. **Figure S1.** The messy chromatogram from 2 mL of LRS6 strain culture in polypropylene-made deepwell microplates. **Figure S2.** Representative chromatograms of gene dosage study. **Figure S3.** Representative mass spectrum for LRS6 strain cultivated in shake flask. **Figure S4.** Representative chromatograms of the strains expressing self-sufficient *CYP725A4*, *CYP725A4* only and parent taxadiene-producing LRS5, all cultivated in 5 mL of YPG in 10 mL glass tubes. **Figure S5.** Representative chromatograms for BN6, expressing *Taxus CYP725A4* and *POR* in tandem, in different culture media. **Figure S6.** Marginal model plots for design of experiment (DoE) study for all predictors (Flavin Adenine Dinucleotide (FAD), Flavin Mononucleotide (FMN), Riboflavin, Hemin and δ-Aminolevulinic acid (ALA)) and their interactions on: **Figure S7.** DoE study results according to run number. **Figure S8.** Preliminary resting cell assay to test the effect of acid–base on side-product (OCT+ iso-OCT) to main product (T5α-ol) ratio. **Figure S9.** Representative chromatograms for the resting cell assays. **Figure S10.** Representative chromatograms for the resting cell assays with extracted ion of 288 m/z for confirming the identified diterpenoids. **Figure S11.** Representative mass spectra from resting cell assay experiment. **Figure S12.** Representative mass spectra from resting cell assay experiment.

## Data Availability

All data are included in the manuscript and Additional information, and further queries about sharing data and/or yeast strains can be directed to the corresponding author.

## Abbreviations

ALA: δ-Aminolevulinic acid
AxRED: *Albidovulum xiamenense* CYP116B64 Reductase
BHIG: Brain heart infusion broth-2% (w/v) galactose
BMR: *Bacillus megaterium* P450_BM3_ reductase
BTS1: Geranylgeranyl diphosphate synthase
crtE: Geranylgeranyl diphosphate synthase
CYP725A4: Taxadiene-5α-hydroxylase
DoE: Design of experiment
DMAPP: Dimethylallyl pyrophosphate
DSD: Definitive screening design
ERG8: Phosphomevalonate kinase
ERG9: Farnesyl-diphosphate farnesyl transferase (squalene synthase)
ERG10: 3-Hydroxy-3-methylglutaryl-CoA (HMG-CoA) synthase
ERG12: Mevalonate kinase
ERG13: 3-Hydroxy-3-methylglutaryl-CoA (HMG-CoA) synthase
ERG19: Mevalonate pyrophosphate decarboxylase
ERG20: Farnesyl pyrophosphate synthetase
FAD: Flavin adenine dinucleotide
fldA: *Escherichia coli* Flavodoxin
FMN: Flavin mononucleotide
FPP: Farnesyl diphosphate
Fpr: *Escherichia coli* Flavodoxin reductase
GGOH: (E,E,E)-geranylgeraniol
GGPP: (E,E,E)-Geranylgeranyl diphosphate
GPP: Geranyl diphosphate
HMG1: 3-Hydroxy-3-methylglutaryl-coenzyme A reductase 1
HMG2: 3-Hydroxy-3-methylglutaryl-coenzyme A reductase 2
IDI: Isopentenyl-diphosphate delta-isomerase
IPP: Isopentenyl pyrophosphate
iso-OCT: 5(11)-Oxa-3(11)-cyclotaxane
LBG: LB broth- 2% (w/v) galactose
mvaE: Acetyl-CoA acetyltransferase/HMG-CoA reductase
mvaS: Hydroxymethylglutaryl-CoA synthase
OCT: 5(12)-Oxa-3(11)-cyclotaxane
POR: *Taxus cuspidata* Cytochrome P450 reductase
RhFRED: *Rhodococcus* Sp. P450_RhF_ Reductase
ROS: Reactive oxygen species
SDG: Synthetic defined-2% (w/v) galactose
T5α-ol: Taxadiene-5α-ol
TASY: Taxadiene synthase
TAT: Taxa-4(20),11(12)-dien-5α-olO-acetyl transferase
TBMG: Terrific broth, modified-2% (w/v) galactose
YPG: Yeast extract peptone-2% (w/v) galactose
