# Supplementary material for: Exploring optimal Taxol® CYP725A4 activity in Saccharomyces cerevisiae
Source: Microb Cell Fact. 2022 Sep 19;21:197. doi: 10.1186/s12934-022-01922-1 (PMC9484169; doi:10.1186/s12934-022-01922-1)
Supplement: Supplementary file 1 — Additional file 1: Table S1. Schematic representation of strains in this study. Table S2. List of primers used in the study. Table S3. Sequence of genes in this study. Table S4. List of guide RNA sequences used in this study. Table S5. List of linkers used in this study. Table S6. Augmented definitive screening designs for optimising P450-reductase expressions, including controls. Table S7. Linear model formulas for response factors in definitive screening designs. Figure S1. The messy chromatogram from 2 mL of LRS6 strain culture in polypropylene-made deepwell microplates. Figure S2. Representative chromatograms of gene dosage study. Figure S3. Representative mass spectrum for LRS6 strain cultivated in shake flask. Figure S4. Representative chromatograms of the strains expressing self-sufficient CYP725A4, CYP725A4 only and parent taxadiene-producing LRS5, all cultivated in 5 mL of YPG in 10 mL glass tubes. Figure S5. Representative chromatograms for BN6, expressing Taxus CYP725A4 and POR in tandem, in different culture media. Figure S6. Marginal model plots for design of experiment (DoE) study for all predictors (Flavin Adenine Dinucleotide (FAD), Flavin Mononucleotide (FMN), Riboflavin, Hemin and δ-Aminolevulinic acid (ALA)) and their interactions on: Figure S7. DoE study results according to run number. Figure S8. Preliminary resting cell assay to test the effect of acid–base on side-product (OCT+ iso-OCT) to main product (T5α-ol) ratio. Figure S9. Representative chromatograms for the resting cell assays. Figure S10. Representative chromatograms for the resting cell assays with extracted ion of 288 m/z for confirming the identified diterpenoids. Figure S11. Representative mass spectra from resting cell assay experiment. Figure S12. Representative mass spectra from resting cell assay experiment. [file 12934_2022_1922_MOESM1_ESM.docx]

Additional Table 1. **Schematic representation of strains in this study.** Sources for reductase coding sequences: POR, Taxus cuspidata; EcFldA and EcFpr, E. coli flavodoxin and flavodoxin reductase; AxRED, Albidovulum xiamenense CYP116B64; RhFRED, Rhodococcus sp. P450_RhF_; BMR, Bacillus megaterium P450_BM3_. The “L” represents linker as listed in Additional Table 5. The lines on “POR” of BNF-8 show that it is in truncated form (without transmembrane sequence domain).

| **Strain Name** | **Schematic Representation of Chromosomally-Expressed Gene Cassettes** |
| --- | --- |
| Synthetic Biology Open Language Symbols | 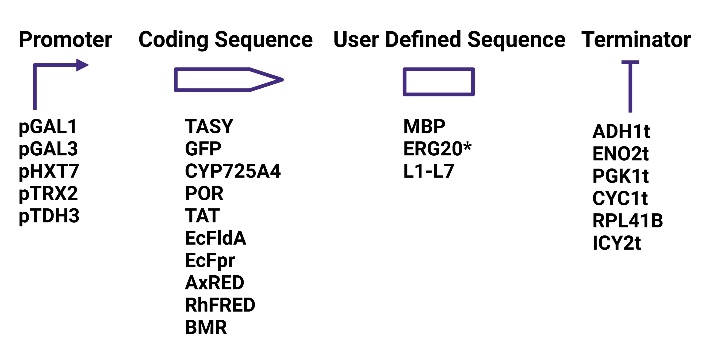 |
| LRS5 | 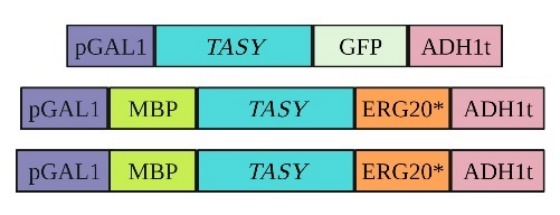 |
| LRS6  [Parent Strain: LRS5] | 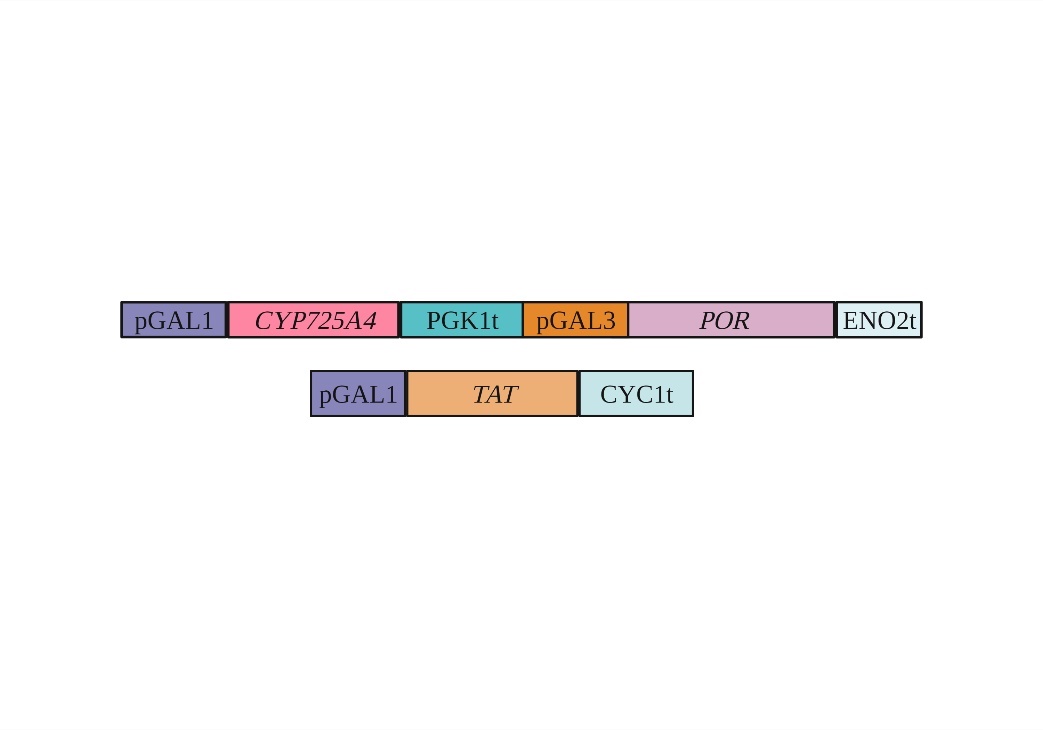 |
| BN1  [Parent Strain: LRS6] | 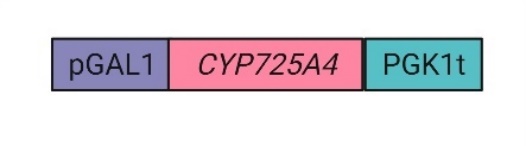 |
| BN2  [Parent Strain: LRS6] | 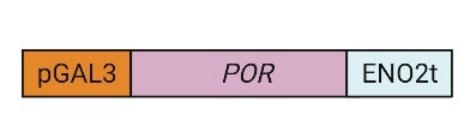 |
| BN3  [Parent Strain: LRS5] | 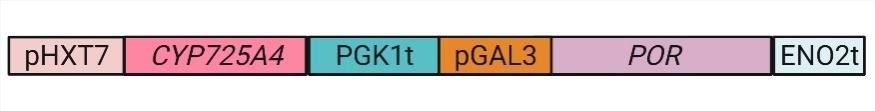 |
| BN4  [Parent Strain: LRS6] | 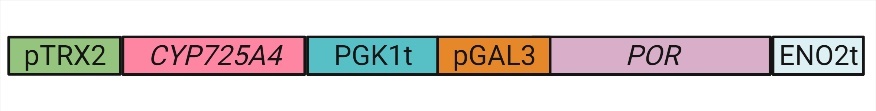 |
| BN5  [Parent Strain: LRS5] | 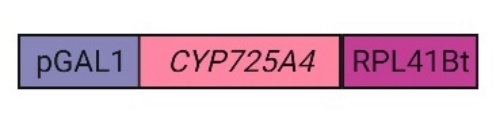 |
| BN6  [Parent Strain: LRS5] | 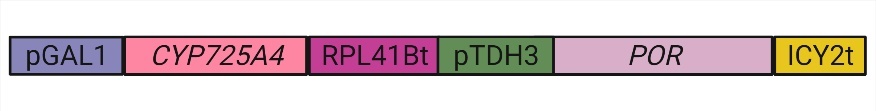 |
| BNF-1  [Parent Strain: LRS5] | 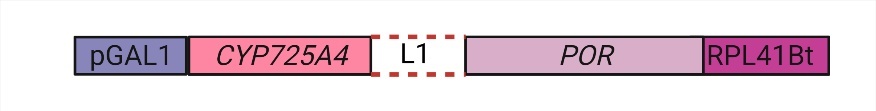 |
| BNF-2  [Parent Strain: LRS5] | 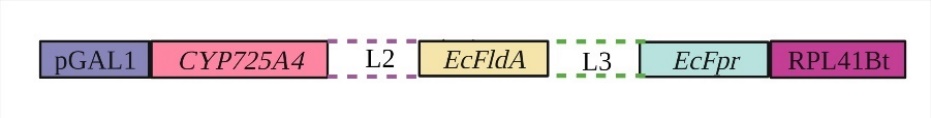 |
| BNF-3  [Parent Strain: LRS5] | 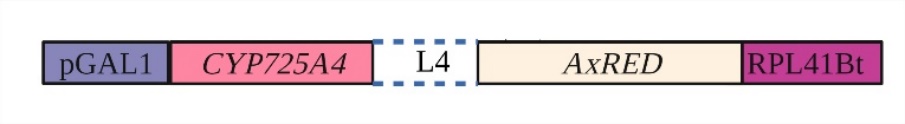 |
| BNF-4  [Parent Strain: LRS5] | 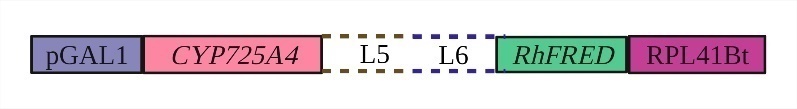 |
| BNF-5  [Parent Strain: LRS5] | 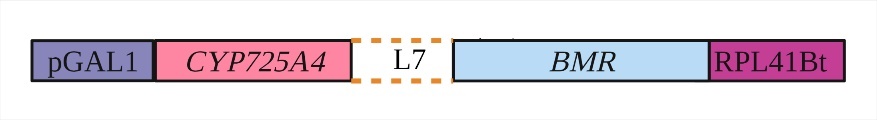 |
| BNF-6  [Parent Strain: LRS5] | 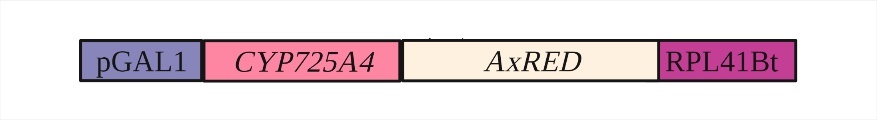 |
| BNF-7  [Parent Strain: LRS5] | 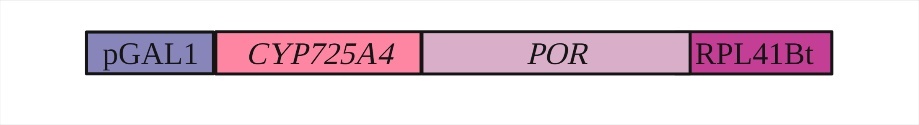 |
| BNF-8  [Parent Strain: LRS5] | 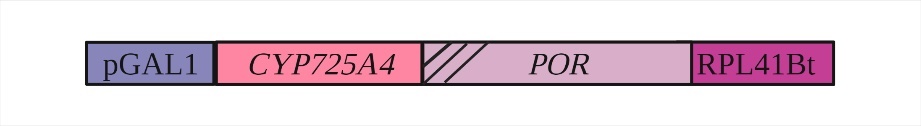 |

Additional Table 2. **List of primers used in the study**

| **Primer** | **Sequence** | **Usage** |
| --- | --- | --- |
| TICY2-[DOWNX2]-F_2 | CTATACTCAACCCGCCCCCAATGCTGAAAATTGTTTGGTGCAAATTTGAAGTCAG | Donor DNA/BN6 |
| TICY2-[DOWNX2]-R_2 | CAAATCATGGACTAGATGAA | Donor DNA/BN6 |
| *CPR-[TICY2]-DOWNX2-F* | AAATGGACGGTCGTTACTTGAGAGACATCTGGTAACGCATTTTACCACATCTCTA | Donor DNA/BN6 |
| *CPR-[TICY2]-DOWNX2-R* | ATCAACAGACCTAAGCTGACTTCAAATTTGCACCAAACAATTTTCAGCATTGGGG | Donor DNA/BN6 |
| *PTDH3-[CPR]-TICY2-F* | ACTTAGTTTCGAATAAACACACATAAACAAACAAAATGCAAGCTAACTCTAATAC | Donor DNA/BN6 |
| *PTDH3-[CPR]-TICY2-R* | GGTCGTGAGAACAAGTAGAGATGTGGTAAAATGCGTTACCAGATGTCTCTCAAGT | Donor DNA/BN6 |
| CYP-[RPBT]-PTHD-F_2 | AGGGTTTCTCTATTAAGTTGTTCCCAAGACCATAAGCGGATTGAGAGCAAATCG | Donor DNA/BN6 |
| CYP-[RPBT]-PTHD-R_2 | GAAATGGCAGTATTGATAATGATAAACTCGAACTGAGCCGAAAATCTTTCAAGC | Donor DNA/BN6 |
| [PTDH3]-CPR-F | TGAATCTTTCTGTCGTGCTTGAAAGATTTTCGGCTCAGTTCGAGTTTATCATTAT | Donor DNA/BN6 |
| [PTDH3]-CPR-R | GGAAGCACCTTCGACGGTATTAGAGTTAGCTTGCATTTTGTTTGTTTATGTGTGTT | Donor DNA/BN6 |
| [UPX2]-pGAL1_f | TACGTTCCACTACGAGGCCA | Donor DNA/ BN5 and BN6 |
| [UPX2]- pGAL1 _r | AGAGAAGGTTTTTTTAGGCTAAGATAATGGGGAGAAAAGAGCTCAAAGAGAAAGAGC | Donor DNA/ BN5 and BN6 |
| UPX2-[ pGAL1]-F | GAGACCAGCTCTTTCTCTTTGAGCTCTTTTCTCCCCATTATCTTAGCCTAAA | Donor DNA/ BN5 and BN6 |
| UPX2-[ pGAL1]-R | TTAGCGACAGTAGACTTGTACAAAGCGTCCATTATAGTTTTTTCTCCTTGACG | Donor DNA/ BN5 and BN6 |
| [CYP]-RPL41B-F | CCTCTATACTTTAACGTCAAGGAGAAAAAACTATAATGGACGCTTTGTACAAGTC | Donor DNA/BN5 and BN6 |
| [CYP]-RPL41B-R | CTTGACCTGAACTTAACGATTTGCTCTCAATCCGCTTATGGTCTTGGGAACAACT | Donor DNA/ BN5 and BN6 |
| [RPBT]-DOWNX2-F | AGGGTTTCTCTATTAAGTTGTTCCCAAGACCATAAGCGGATTGAGAGCAAATCGT | Donor DNA/BN5 |
| CPR-[RPBT]-DNX2-R | ATCAACAGACCTAAGCTGACTTCAAATTTGCACCAAGCCGAAAATCTTTCAAGCA | Donor DNA/BN5 |
| RPBT-[DOWNX2]-F | TGAATCTTTCTGTCGTGCTTGAAAGATTTTCGGCTTGGTGCAAATTTGAAGTCAG | Donor DNA/BN5 |
| RPBT-[DOWNX2]-R | CAAATCATGGACTAGATGAA | Donor DNA/BN5 |
| BNF2_CYP.FOR | GAAAAAACTATAATGGACGCTTTGTACAAGTCTACT | Gibson Assembly |
| BNF2_CYP.REV | CAGCACCAGAAGCTGGTCTTGGGAACAAC | Gibson Assembly |
| BNF2_L20WANG.FOR | TTCCCAAGACCAGCTTCTGGTGCTGG | Gibson Assembly |
| BNF2_L20WANG.REV | ACACTACTTTAGAAGTAGCACCAGAAGT | Gibson Assembly |
| BNF1_8_ pGAL1.FOR | ACGACGGCCAGTCCCCATTATCTTAGCCTAAAAAAACCTTC | Gibson Assembly |
| BNF1_8_ pGAL1.REV | ACAAAGCGTCCATTATAGTTTTTTCTCCTTG | Gibson Assembly |
| BNF2_RPBt.FOR | AGTAGTGCCTGAGCGGATTGAGAG | Gibson Assembly |
| BNF2_RPBt.REV | CCATGATTACGCCAGCCGAAAATCTTTCAAGCACG | Gibson Assembly |
| pUC19_FINAL.FOR | AGATTTTCGGCTGGCGTAATCATGGTCATAGCTGT | Gibson Assembly |
| pUC19_FINAL.REV | TAAGATAATGGGGACTGGCCGTCGTTTTACAAC | Gibson Assembly |
| BNF2_FLDA.FOR | TCTGGTGCTACTGCTATCACTGGC | Gibson Assembly |
| BNF2_FLDA.REV | TGGTGGCTCCATGGGCATTGAGAATTTCG | Gibson Assembly |
| BNF2_FPR.FOR | AACCGCCGCCACCGCTGATTGGGTA | Gibson Assembly |
| BNF2_FPR.REV | GCTCTCAATCCGCTTACCAGTAATG | Gibson Assembly |
| BNF2_P4BAKL.FOR | ATTCTCAATGCCCATGGAGCCACCACCG | Gibson Assembly |
| BNF2_P4BAKL.REV | TTACCCAATCAGCGGTGGCGGCGGT | Gibson Assembly |
| BNF2_RPBt.FOR | CATTACTGGTAAGCGGATTGAGAGCA | Gibson Assembly |
| BNF2_Wang20L.FOR | TTCCCAAGACCAGCTTCTGGTGCTGGT | Gibson Assembly |
| BNF2_Wang20L.REV | TGCCAGTGATAGCAGTAGCACCAGA | Gibson Assembly |
| CPR_BNF8.FOR | GGTGCTTTTAGACGTAGAGGTGGT | Gibson Assembly |
| CPR_ BNF8.REV | GCTCTCAATCCGCTTACCAGATGTC | Gibson Assembly |
| CYP_ BNF8andBNF1.FOR | GAAAAAACTATAATGGACGCTTTGTACAAGTCTACT | Gibson Assembly |
| CYP_ BNF8and BNF1.REV | TAGAAACATCCATTGGTCTTGGGAACA | Gibson Assembly |
| DVSL_ BNF8.FOR | TTCCCAAGACCAATGGATGTTTCTACT | Gibson Assembly |
| DVSL_ BNF8.REV | AACCACCTCTACGTCTAAAAGCACC | Gibson Assembly |
| CPR_ BNF1.FOR | GGTGCTTTTAGACAAGCTAACTCTAATACCG | Gibson Assembly |
| CPR_ BNF1.REV | GCTCTCAATCCGCTTACCAGATGTC | Gibson Assembly |
| DVSL_ BNF1.FOR | TTCCCAAGACCAATGGATGTTTCTACT | Gibson Assembly |
| DVSL_ BNF1.REV | TAGAGTTAGCTTGTCTAAAAGCACCCA | Gibson Assembly |
| BNF-1-RPBt_new.for | CATCTGGTAAGCGGATTGAGAGCA | Gibson Assembly |
| BNF-1-RPBt_new.rev | TGATTACGCCAGCCGAAAATCT | Gibson Assembly |
| BNF-1-CPR_new.for | TGCTTTTAGACAAGCTAACTCTAATACCGT | Gibson Assembly |
| BNF-1-CPR_new.rev | CTCAATCCGCTTACCAGATGTCT | Gibson Assembly |
| CYP_BNF-3.FOR | GAAAAAACTATAAATGGACGCTTTGTACAAGTCTACTGT | Gibson Assembly |
| CYP_BNF-3.REV | ATACCGCTCTCTCTGGTCTTGGGAACAACTTAATAGAGAAACC | Gibson Assembly |
| RPBt_BNF-3, 6.FOR | CTGGGCTTATGAGCGGATTGAGAGCAAATCGT | Gibson Assembly |
| RPBt_BNF-3.REV | CGAGCTCGAATTCAGCCGAAAATCTTTCAAGCACG | Gibson Assembly |
| XIAR_BNF-3.FOR | TTCCCAAGACCAGAGAGAGCGGTATCATTCCCGATCG | Gibson Assembly |
| XIAR_BNF-3, 6.REV | GCTCTCAATCCGCTCATAAGCCCAGGACGATCCTACGCCCC | Gibson Assembly |
| CYP_BNF-4, 5, 6, 7.FOR | GAAAAAACTATAATGGACGCTTTGTACAAGTCTACTG | Gibson Assembly |
| CYP_BNF-4.REV | TCATGTGCGTTGATGGTCTTGGGAACAACTTAATAGAGAAAC | Gibson Assembly |
| RHFLL_BNF-4.FOR | TTCCCAAGACCATCAACGCACATGAGGCTTGC | Gibson Assembly |
| RHFLL_BNF-4.REV | GCTCTCAATCCGCTCAGCACAACCTCAAAGCTAATCTATCAC | Gibson Assembly |
| RPBt_BNF-4.FOR | AGGTTGTGCTGAGCGGATTGAGAGCAAATCGT | Gibson Assembly |
| RPBt_BNF-4, 5, 6, 7.REV | CCATGATTACGCCAGCCGAAAATCTTTCAAGCACGACAG | Gibson Assembly |
| BM3LL_BNF-5.FOR | TTCCCAAGACCAATGGGTGGCATACCATCGC | Gibson Assembly |
| BM3LL_BNF-5.REV | GCTCTCAATCCGCCTAACCGGCCCATACATCCT | Gibson Assembly |
| CYP_BNF-5.REV | GTATGCCACCCATTGGTCTTGGGAACAACTTAATAGAGAAACCCT | Gibson Assembly |
| RPBt_BNF-5.FOR | ATGGGCCGGTTAGGCGGATTGAGAGCAAATCGT | Gibson Assembly |
| AXIAR_BNF-6.FOR | TTCCCAAGACCAATGATCCTAAGGCGTGTCGT | Gibson Assembly |
| CYP_BNF-6.REV | GCCTTAGGATCATTGGTCTTGGGAACAACTTAATAGAGAAACC | Gibson Assembly |
| CYP_BNF-8.FOR | GAAAAAACTATAATAAATGGACGCTTTGTACAAGTCTACTG | Gibson Assembly |
| CYP_BNF-8.REV | AACCACCTCTACGGGTCTTGGGAACAACTTAATAGAGAAACC | Gibson Assembly |
| tCPR_BNF-8.FOR | GTTCCCAAGACCCGTAGAGGTGGTTCTGATACTCAAAAGC | Gibson Assembly |
| (t)CPR_BNF-7, 8.REV | GCTCTCAATCCGCTTACCAGATGTCTCTCAAGTAACGACCG | Gibson Assembly |
| RPBt_BNF-7, 8.FOR | GACATCTGGTAAGCGGATTGAGAGCAAATCGT | Gibson Assembly |
| RPBt_BNF-8.REV | GAGCTCGAATTCAAGCCGAAAATCTTTCAAGCACG | Gibson Assembly |
| CPR_BNF-7.FOR | TTCCCAAGACCAATGCAAGCTAACTCTAATACCGTCG | Gibson Assembly |
| CYP_BNF-7.REV | AGTTAGCTTGCATTGGTCTTGGGAACAACTTAATAGAGAAACC | Gibson Assembly |
| DVSJohnsonLinker+ | GATGTTTCTACTGAACAATCTGCTAAAGAAGCTCCAGCTGAAACTTTGGGTGCTTTTAGA | Linker Sequence |
| DVSJohnsonLinker- | CTACAAAGATGACTTGTTAGACGATTTCTTCGAGGTCGACTTTGAAACCCACGAAAATCT | Linker Sequence |
| 20aaWangLinker+ | GCTTCTGGTGCTGGTGGTTCTGAAGGTGGTGGTTCTGAAGGTGGTACTTCTGGTGCTACT | Linker Sequence |
| 20aaWangLinker- | CGAAGACCACGACCACCAAGACTTCCACCACCAAGACTTCCACCATGAAGACCACGATGA | Linker Sequence |
| P4BAKKESLinker+ | CATGGAGCCACCACCGCCGCTACCGCCGCCACCGCTGCCACCACCGCCAGAACCGCCGCCACC | Linker Sequence |
| P4BAKKESLinker- | GTACCTCGGTGGTGGCGGCGATGGCGGCGGTGGCGACGGTGGTGGCGGTCTTGGCGGCGGTGG | Linker Sequence |
| pGAL1 -RPBt.for | CAAGAGACCAGCTCTTTCTCTTTGAGCTCTTTTCTCCCCATTATCTTAGCCTAAA | Donor from assembled DNA |
| pGAL1 -RPBt.rev | ATCAACAGACCTAAGCTGACTTCAAATTTGCACCAAGCCGAAAATCTTTCAAGCA | Donor from assembled DNA |
| ColPCR_upX2_pGAL1 _F | TTTCGTCTTCTAGCTTAGCG | Colony PCR |
| ColPCR_upX2_ pGAL1 _R | CAGCGATAGCGGATAAAGCG | Colony PCR |
| ColPCR/SeqFusion_RPBTDownX2_F | GCAAATCGTTAAGTTCAGGTC | Colony PCR |
| ColPCR/SeqFusion_RPBTDownX2_R | TCTCGTCCAACTTCCATCAA | Colony PCR/Sequencing |
| For_Up416(TRX2) | TATCGTCCAACTGCATGGAG | Donor DNA/Gene Dosage |
| Rev_Up416(TRX2) | TTGTTTATTTTTGACCAGGAGCAATAAGAGGGGTCCGGTTAAACGGATCT | Donor DNA/Gene Dosage |
| Rev_PromG(416,1309,SAP155_PGKt) | ATTGATCTATCGATTTCAATTCAATTCAATTTATGGTCTTGGGAACAACT | Donor DNA/Gene Dosage |
| Rev_416Term(PGKt) | GAAGGAGCATGTTCGGCACACAGTGGACCGTAAACTTAAAATACGCTGAA | Donor DNA/Gene Dosage |
| Rev_Down416(PGKt) | GAAACTTGAAAGGTGTGGCCT | Donor DNA/Gene Dosage |
| For_Down416(PGK1) | TATGTTCGGGTTCAGCGTATTTTAAGTTTACGGTCCACTGTGTGCCGAAC | Donor DNA/Gene Dosage |
| For_[PromG]_PGK1/1309, 416d | AAGAAACTTACTATGACGCAGTTTAGGATCAGTACGGATTAGAAGCCGC | Donor DNA/Gene Dosage |
| For_Term1309,416[PGKt] | TTCTCTATTAAGTTGTTCCCAAGACCATAAATTGAATTGAATTGAAATCG | Donor DNA/Gene Dosage |
| For_PTRX2 [416] | CATCAATGCGAGATCCGTTTAACCGGACCCCTCTTATTGCTCCTGGTCAA | Donor DNA/Gene Dosage |
| Rev_PTRX2[416] | AGCGACAGTAGACTTGTACAAAGCGTCCATAAGTCTTCGCCTAAAATTAA | Donor DNA/Gene Dosage |
| For_Gene (416and511/TRX2/PGKT) | TACCAGTGAGTTAATTTTAGGCGAAGACTTATGGACGCTTTGTACAAGTC | Donor DNA/Gene Dosage |
| For_Up511(HXT7, CAS) | TTTGCACATAAAGGGTGCC | Donor DNA/Gene Dosage |
| Rev_Up511(HXT7,CAS) | GTAAATTCTGTTAATCAAAGAAAAAGCAATGAAATCTGTACCAACCGTAT | Donor DNA/Gene Dosage |
| For_HXT7(511/CAS) | TCTTTCACCTATACGGTTGGTACAGATTTCATTGCTTTTTCTTTGATTAAC | Donor DNA/Gene Dosage |
| Rev_HXT7 (511/Cas) | AGCGACAGTAGACTTGTACAAAGCGTCCATTGTACAAGAGAATGTTCAGC | Donor DNA/Gene Dosage |
| Rev_Gene+PGKT(511andPTRX(416, 511)) | GATTACGAGGATACGGAGAGAGGTATGTACTAAACTTAAAATACGCTGAACC | Donor DNA/Gene Dosage |
| For_Gene+PGKt(511_HXT7) | ATTATTTGGTGCTGAACATTCTCTTGTACAATGGACGCTTTGTACAAGTC | Donor DNA/Gene Dosage |
| For_Gal+CPR (511/enot) | TATGTTCGGGTTCAGCGTATTTTAAGTTTAGTACATACCTCTCTCCGTAT | Donor DNA/Gene Dosage |
| Rev_Gal+CPR(511/ENOT) | AAAGACTAATAATTCTTAGTTAAAAGCACTTTACCAGATGTCTCTCAAGT | Donor DNA/Gene Dosage |
| Rev_Enot(511) | AAGAAAATAGAAGCAAACGACGTAATGCCGATGTTTTCTTATCATCCATG | Donor DNA/Gene Dosage |
| For_Enot(511) | GACGGTCGTTACTTGAGAGACATCTGGTAAAGTGCTTTTAACTAAGAATT | Donor DNA/Gene Dosage |
| For_Down511(Enot) | AAAATAACTACATGGATGATAAGAAAACATCGGCATTACGTCGTTTGCTT | Donor DNA/Gene Dosage |
| Rev_Down511(Enot) | TAACAACATAGCGGCAGCTGC | Donor DNA/Gene Dosage |
| For_PromG_511/TRX | TCTTTCACCTATACGGTTGGTACAGATTTCCTCTTATTGCTCCTGGTCAA | Donor DNA/Gene Dosage |
| Rev_PromG_511/TRX | ATTGATCTATCGATTTCAATTCAATTCAATTTATGGTCTTGGGAACAACT | Donor DNA/Gene Dosage |
| F_UpH(416d) | TATCGTCCAACTGCATGGAGATGAGTCGTG | Donor DNA/Gene Dosage |
| R_UpH(416d) | TCGCCCGCTCGGCGGCTTCTAATCCGTACTGGGTCCGGTTAAACGGATCTCGCA | Donor DNA/Gene Dosage |
| F_Cas(416d) | CATCAATGCGAGATCCGTTTAACCGGACCCAGTACGGATTAGAAGCCGCC | Donor DNA/Gene Dosage |
| R_Cas(416d) | GAAGGAGCATGTTCGGCACACAGTGGACCGCTTCGAGCGTCCCAAAACCT | Donor DNA/Gene Dosage |
| F_DownH(416d) | TTGCTTGAGAAGGTTTTGGGACGCTCGAAGCGGTCCACTGTGTGCCGAACA | Donor DNA/Gene Dosage |
| R_DownH(416d) | GAAACTTGAAAGGTGTGGCCTTTAGTTTTG | Donor DNA/Gene Dosage |
| ColonyP_F_UpH(416d) | TATTAACCGCTTTTACTATTATC | Colony PCR/Gene Dosage |
| ColonyP_R_DownH(416d) | ATATCCACATCAATGGCTAA | Colony PCR/Gene Dosage |
| ColonyP_R_PGal(416d) | GATCAAAAATCATCGCTTCG | Colony PCR/Gene Dosage |
| ColonyP_F_GeneTerm(416d) | GTTGTTGTGGACTACTTACT | Colony PCR/Gene Dosage |
| ColPCR_416d_f | ATCTTCTACGCTGACAGTAA | Colony PCR/Gene Dosage |
| ColPCR_416d_r_1 | CCGTTCAACAATAAAGCGAA | Colony PCR/Gene Dosage |
| ColPCR_416d_r_2 | AATGTGGTAACAAAGGTGTT | Colony PCR/Gene Dosage |
| ColPCR_trx_416d_f_1 | AATTTTGTTGTTCGTCCACC | Colony PCR/Gene Dosage |
| ColPCR_trx_416d_r_1 | TGGCGATTTCATTCTTTCAA | Colony PCR/Gene Dosage |
| ColPCR_trx_416d_f_2 | ACCATCAGCCTCAAGTCGTC | Colony PCR/Gene Dosage |
| ColPCR_trx_416d_r_2 | ATCTCTGACCGGAGAACTGA | Colony PCR/Gene Dosage |
| ColPCR_511b_f_1 | TGAGCATACCCGCCTTTTC | Colony PCR/Gene Dosage |
| ColPCR_511b_f_2 | CTTCTAAGGCTGAGTTTTTGGTTAAGA | Colony PCR/Gene Dosage |
| ColPCR_511b_r | TTTTTCTCTGTCAAGGGACA | Colony PCR/Gene Dosage |
| Seq_BN2_f | GAGGGTGCTACCAAGGAATAC | Sequencing/Gene Dosage |
| Seq_BN2_r | GCACTCCTGATTCCGCTAATA | Sequencing/Gene Dosage |
| SeqBN3 FWD Set 1 | CCTATTAGCGGAATCAGGAGTG | Sequencing/Gene Dosage |
| SeqBN3(SET2) FWD | ACGGGCGATGAGTAAGAAAG | Sequencing/Gene Dosage |
| SeqBN3(SET2) REV | GGGTGACCAATCAAGGAAGT | Sequencing/Gene Dosage |
| SeqBN4 (SET1) FWD | CCTATTAGCGGAATCAGGAGTG | Sequencing/Gene Dosage |
| SeqBN4 (SET2) REV | TTGCAGGCGGTCAATCAA | Sequencing/Gene Dosage |
| pWS082_Linear-F | ATCAACAACAGAGGACATATG | gRNA Vector |
| pWS082_Linear-R | ATCCTGCACTCATCTACTACC | gRNA Vector |
| pWS158_Linear-F | GTTTTAGAGCTAGAAATAGCAAGT | CRISPR/Cas9 Vector |
| pWS158_Linear-R | AGTCCCATTCGCCACCC | CRISPR/Cas9 Vector |
| p426_Cas9_gRNA-ARS511b_F (+511b gRNA sequence) | GTTTTAGAGCTAGAAATAGCAAGTTAAAATAAGGCTAGTCCG | gRNA/CRISPR Vector |
| p426_Cas9_gRNA-ARS511b_R (+511b gRNA sequence) | AAAGTCCCATTCGCCACCCGAAGG | gRNA/CRISPR Vector |
| 416d gRNA scaffold | GCAACACCTTCGGGTGGCGAATGGGACTTTTAGTGCACTTACCCCACGTTGTTTTAGAGCTAGAAATAGCAAGTTAAAAT | The green labelling denotes the homology sequences for integration of sgRNA sequence (in magenta) into linearised p426_Cas9_gRNA-ARS511b vector without 511b targeting sequence) |

Additional Table 3. **Sequence of genes used in this study**

| **Gene and Source** | **Sequence** |
| --- | --- |
| Codon Optimised  *Taxus cuspidata CYP725A4*  (1) | atggacgctttgtacaagtctactgtcgctaaattcaacgaagtcactcaattagattgttctactgaatctttttccatcgctttatccgctatcgctggtatcttgttgttattattgttgttcagatctaagagacattcctctttgaaattgcctccaggtaagttaggtatcccattcattggtgagtccttcatcttcttgagagctttgagatccaactctttagaacaatttttcgacgaaagagttaagaagtttggtttggtcttcaagacttccttgattggtcacccaactgttgttttgtgcggtccagctggtaacagattaatcttgtctaatgaagaaaagttagttcaaatgtcttggccagcccaattcatgaagttgatgggtgaaaactctgtcgctaccagacgtggtgaagatcacattgttatgagatctgctttggctggttttttcggtccaggtgctttacaatcctacatcggtaagatgaacactgaaattcaatctcacattaacgaaaaatggaagggtaaggatgaagttaacgtcttgccattggtcagagaattggtcttcaatatttctgctatcttattcttcaacatctacgacaagcaagagcaagatcgtttacataagttattggaaactattttggttggttctttcgctttgccaattgatttacctggttttggttttcacagagctttgcaaggtagagctaagttgaacaagattatgttatctttaatcaagaagcgtaaggaagatttgcaatccggttccgctactgctactcaagacttgttatccgtcttgttgaccttccgtgacgacaagggtactccattgactaacgacgaaattttagacaatttctcctctttattacacgcttcctacgacactaccacctccccaatggctttgatctttaagttgttgtcctccaaccctgaatgttatcaaaaggtcgttcaagaacaattggaaattttgtccaataaagaagaaggtgaggaaatcacttggaaagatttgaaggctatgaaatatacctggcaagttgctcaagaaaccttacgtatgttcccaccagtttttggtaccttcagaaaggctattaccgacattcaatacgacggttacactattccaaagggttggaagttgttgtggactacttactctactcacccaaaggatttgtactttaacgaacctgaaaaatttatgccatccagatttgaccaagaaggtaagcacgtcgccccatataccttcttgccattcggtggtggtcaaagatcctgcgttggttgggaattctctaaaatggaaattttgttgttcgtccaccacttcgtcaaaactttctcctcctatacccctgttgacccagacgaaaagatctctggtgatccattgccaccattgccatctaagggtttctctattaagttgttcccaagaccataa |
| Codon Optimised  *Taxus cuspidata POR* (+transmembrane domain prediction by THMM (2))  (1) | atgcaagctaactctaataccgtcgaaggtgcttcccaaggtaaatccttgttagatatttctagattggaccatatcttcgctttattgttgaacggtaagggtggtgacttgggtgctatgaccggttctgctttgattttgactgaaaactctcaaaacttaatgattttgaccaccgccttagccgttttggttgcctgtgtcttcttcttcgtttggcgtagaggtggttctgatactcaaaagcctgccgtcagacctactcctttggttaaggaagaagacgaagaagaagaagatgattccgctaagaagaaggtcactattttttttggtacccaaaccggtactgctgaaggtttcgccaaagctttggctgaagaagctaaggccagatacgaaaaggccgtttttaaggtcgttgacttggataactacgctgctgatgatgaacaatacgaagagaagttgaaaaaggaaaagttggctttctttatgttggctacctatggtgacggtgaaccaaccgataacgccgctcgtttctacaagtggttcttggaaggtaaagaacgtgaaccatggttgtctgacttgacttacggtgttttcggtttgggtaacagacaatacgaacactttaacaaggtcgccaaggctgttgacgaagttttaatcgaacaaggtgctaagagattggttccagtcggtttgggtgacgacgatcaatgcattgaggatgatttcactgcttggagagaacaagtttggccagaattggatcaattgttgagagatgaagacgacgaaccaacttctgctaccccatacaccgctgctatccctgaatacagagttgaaatttacgactccgttgtttctgtttacgaagaaactcacgccttgaagcaaaacggtcaagctgtctacgacattcaccatccatgtcgttctaatgtcgctgttagaagagaattgcatactccattatctgatagatcctgtatccacttggaattcgacatctctgataccggtttgatctacgaaactggtgaccacgttggtgtccacactgaaaactctattgaaaccgttgaagaagccgccaagttgttgggttaccaattggatactatcttctctgtccacggtgacaaggaagacggtactccattgggtggttcttctttgcctcctccattcccaggtccatgtactttgagaactgctttggctagatacgccgacttattgaacccaccacgtaaggctgcttttttggctttagccgcccatgcctccgatcctgctgaggctgaaagattgaaatttttgtcttccccagctggtaaggacgaatactcccaatgggttactgcctcccaacgttccttgttggaaattatggctgaattcccatctgctaagccaccattgggtgtcttcttcgctgctattgccccaagattgcaaccacgttactactccatttcctcttctccaagattcgctccatctagaattcacgtcacctgcgctttggtttacggtccatctccaaccggtcgtatccacaaaggtgtttgctctaactggatgaagaactctttgccatccgaagaaactcacgactgttcttgggctccagttttcgtcagacaatctaatttcaaattgccagctgattctactacccctatcgtcatggtcggtcctggtaccggtttcgctccatttagaggttttttgcaagaaagagctaagttgcaagaagctggtgaaaaattgggtcctgccgttttattcttcggttgtagaaacagacaaatggactacatctacgaagatgaattaaagggttatgtcgaaaagggtattttgactaacttaatcgtcgccttctccagagagggtgctaccaaggaatacgttcaacacaaaatgttggaaaaagcttctgatacttggtctttgatcgctcaaggtggttacttgtacgtttgtggtgatgccaaaggtatggctagagatgtccacagaaccttgcataccattgtccaagaacaagaatctgtcgactcttctaaggctgagtttttggttaagaagttacaaatggacggtcgttacttgagagacatctggtaa |
| Codon Optimised  Truncated *Taxus cuspidata* *POR*  (- all transmembrane domain sequences and all other upstream nucleotide sequences)  This Study | cgtagaggtggttctgatactcaaaagcctgccgtcagacctactcctttggttaaggaagaagacgaagaagaagaagatgattccgctaagaagaaggtcactattttttttggtacccaaaccggtactgctgaaggtttcgccaaagctttggctgaagaagctaaggccagatacgaaaaggccgtttttaaggtcgttgacttggataactacgctgctgatgatgaacaatacgaagagaagttgaaaaaggaaaagttggctttctttatgttggctacctatggtgacggtgaaccaaccgataacgccgctcgtttctacaagtggttcttggaaggtaaagaacgtgaaccatggttgtctgacttgacttacggtgttttcggtttgggtaacagacaatacgaacactttaacaaggtcgccaaggctgttgacgaagttttaatcgaacaaggtgctaagagattggttccagtcggtttgggtgacgacgatcaatgcattgaggatgatttcactgcttggagagaacaagtttggccagaattggatcaattgttgagagatgaagacgacgaaccaacttctgctaccccatacaccgctgctatccctgaatacagagttgaaatttacgactccgttgtttctgtttacgaagaaactcacgccttgaagcaaaacggtcaagctgtctacgacattcaccatccatgtcgttctaatgtcgctgttagaagagaattgcatactccattatctgatagatcctgtatccacttggaattcgacatctctgataccggtttgatctacgaaactggtgaccacgttggtgtccacactgaaaactctattgaaaccgttgaagaagccgccaagttgttgggttaccaattggatactatcttctctgtccacggtgacaaggaagacggtactccattgggtggttcttctttgcctcctccattcccaggtccatgtactttgagaactgctttggctagatacgccgacttattgaacccaccacgtaaggctgcttttttggctttagccgcccatgcctccgatcctgctgaggctgaaagattgaaatttttgtcttccccagctggtaaggacgaatactcccaatgggttactgcctcccaacgttccttgttggaaattatggctgaattcccatctgctaagccaccattgggtgtcttcttcgctgctattgccccaagattgcaaccacgttactactccatttcctcttctccaagattcgctccatctagaattcacgtcacctgcgctttggtttacggtccatctccaaccggtcgtatccacaaaggtgtttgctctaactggatgaagaactctttgccatccgaagaaactcacgactgttcttgggctccagttttcgtcagacaatctaatttcaaattgccagctgattctactacccctatcgtcatggtcggtcctggtaccggtttcgctccatttagaggttttttgcaagaaagagctaagttgcaagaagctggtgaaaaattgggtcctgccgttttattcttcggttgtagaaacagacaaatggactacatctacgaagatgaattaaagggttatgtcgaaaagggtattttgactaacttaatcgtcgccttctccagagagggtgctaccaaggaatacgttcaacacaaaatgttggaaaaagcttctgatacttggtctttgatcgctcaaggtggttacttgtacgtttgtggtgatgccaaaggtatggctagagatgtccacagaaccttgcataccattgtccaagaacaagaatctgtcgactcttctaaggctgagtttttggttaagaagttacaaatggacggtcgttacttgagagacatctggtaa |
| Codon Optimised  *Bacillus megaterium* P450_BM3_ Reductase (*BMR*+ natural linker)  (3) and this study | atgggtggcataccatcgcctagtacagaacaaagcgcaaagaaggtcagaaagaaagctgagaatgcacacaacacccccttgctggttttgtatggctccaacatggggacggctgagggtactgctagagacttggcggacatagcgatgtcaaaggggttcgccccacaagttgctactttagactcccatgcaggcaacttgccaagagaaggggcagtcttgatcgtgactgctagttacaatgggcacccgccagacaatgccaaacagttcgtggattggctggatcaggcttccgcagacgaggtcaaaggggtaagatatagtgtcttcggttgtggagacaagaattgggcgacgacataccagaaggttccagcattcattgacgagaccctggctgccaagggtgccgagaatattgcagatcgtggcgaggctgacgcctcagatgatttcgagggtacttacgaggagtggagagagcacatgtggagcgatgttgctgcatatttcaatctagatattgagaattctgaggacaacaagtccaccttaagtttacagttcgttgatagtgcagctgacatgcccttggcaaagatgcatggagctttctccacaaatgtggttgctagtaaagagttgcagcaacccggttccgctagatcaactagacacttggagatagagttacctaaagaggcaagttaccaagagggtgaccacctgggcgtgatccccagaaattacgagggtattgttaatagagtcacggctagatttggtttggacgctagccaacagattagattggaagctgaagaggagaagttggcacacttacctttagcaaagactgtctctgtcgaggaattgttgcagtatgtcgaattgcaggacccagtaactcgtacacaattgagagcgatggccgcaaagactgtatgtccacctcacaaggttgaattggaagcacttttagagaaacaggcatataaagagcaggtcttagcgaagagactaactatgctggagttgttggagaagtatccagcctgcgagatgaagttttctgagttcatagcgttattgccttcaattagacctagatactattctatctcctctagtccaagagttgacgagaagcaggcttctattacagtttctgtagtgtctggtgaggcttggtctggctacggtgagtacaagggtatagcctcaaattacttagcagaattgcaggaaggggacacaataacttgtttcatctctaccccacaaagtgagttcactctaccgaaggacccagagacacccttaattatggttggtccaggtactggagttgctcccttccgtggtttcgtccaagctaggaagcaattgaaggagcagggtcaatccttaggtgaggcccacttgtattttggttgtagatcgccacacgaggattacctataccaagaggaattggagaatgctcagtcagagggaattatcacattacacacagccttcagtagaatgcctaaccaaccaaagacttatgtacaacatgttatggagcaggatggtaagaagttaatagagctgttagaccagggtgctcatttctacatatgtggtgatggttcgcagatggccccagctgtggaagctactctaatgaagtcatacgcagatgtccatcaggttagcgaggctgatgcaagactttggttgcaacaactggaagagaaggggagatatgccaaggatgtatgggccggttag |
| Codon Optimised *Rhodococcus* sp. P450_RhF_ Reductase (*RhFRED* + natural linker) | ctacataggcaccagccagtaacgattggcgagccagctgctagaatggctgtctcgagaactgtgactgtagaacgtttggatagaatagcagatgatgtacttagattggttttgagagatgctggtggtaaaacgctaccaacctggacacctggagctcacattgatttggatttaggtgcccttagcagacaatattctttatgtggtgccccagacgctccttcttatgaaatagcagttcatttagacccagaatcaagaggcgggtccagatatattcatgagcaattagaagtaggctcgccattacgtatgcgtggtccacgtaatcactttgctttagatccgggtgcggaacattatgtctttgttgcaggtggtatagggataacacctgtattggcaatggcagatcatgcgagagctagaggttggtcctatgagttgcattattgtggtagaaatagaagtggtatggcttacctagaaagggtagcaggtcatggcgatagagctgcgttacatgtctcagaagagggtactagaattgatttagcggctttgcttgctgaaccggcgccaggagttcaaatttatgcttgtggaccaggtcgtttactggctgggttggaagatgcttctagaaattggcctgatggagcattgcatgtagaacattttacgtcctcgttggcagcattagaccctgatgttgaacatgcttttgatttggagttgagggattcaggtttgactgtcagggtggagcctactcaaactgtgttagatgcacttagagcgaataatatagatgtaccaagtgattgtgaagagggtctgtgtgggtcttgtgaagtggcagtgttagatggtgaagtggatcacagagatacagttttaacaaaagctgaaagggcagcaaataggcaaatgatgacttgttgttctagggcttgcggtgatagattagctttgaggttgtgctga |
| *Escherichia coli* Flavodoxin (*fldA*)  KEGG entry b0684 (4) | Atggctatcactggcatctttttcggcagcgacaccggtaataccgaaaatatcgcaaaaatgattcaaaaacagcttggtaaagacgttgccgatgtccatgacattgcaaaaagcagcaaagaagatctggaagcttatgacattctgctgctgggcatcccaacctggtattacggcgaagcgcagtgtgactgggatgacttcttcccgactctcgaagagattgatttcaacggcaaactggttgcgctgtttggttgtggtgaccaggaagattacgccgaatatttctgcgacgcattgggcaccatccgcgacatcattgaaccgcgcggtgcaaccatcgttggtcactggccaactgcgggctatcatttcgaagcatcaaaaggtctggcagatgacgaccactttgtcggtctggctatcgacgaagaccgtcagccggaactgaccgctgaacgtgtagaaaaatgggttaaacagatttctgaagagttgcatctcgacgaaattctcaatgcctga |
| *Escherichia coli* Flavodoxin Reductase (*Fpr*)  KEGG entry  b3924 (4) | Atggctgattgggtaacaggcaaagtcactaaagtgcagaactggaccgacgccctgtttagtctcaccgttcacgcccccgtgcttccgtttaccgccgggcaatttaccaagcttggccttgaaatcgacggcgaacgcgtccagcgcgcctactcctatgtaaactcgcccgataatcccgatctggagttttacctggtcaccgtccccgatggcaaattaagcccacgactggcggcactgaaaccaggcgatgaagtgcaggtggttagcgaagcggcaggattctttgtgctcgatgaagtgccgcactgcgaaacgctatggatgctggcaaccggtacagcgattggcccttatttatcgattctgcaactaggtaaagatttagatcgcttcaaaaatctggtcctggtgcacgccgcacgttatgccgccgacttaagctatttgccactgatgcaggaactggaaaaacgctacgaaggaaaactgcgcattcagacggtggtcagtcgggaaacggcagcggggtcgctcaccggacggataccggcattaattgaaagtggggaactggaaagcacgattggcctgccgatgaataaagaaaccagccatgtgatgctgtgcggcaatccacagatggtgcgcgatacacaacagttgctgaaagagacccggcagatgacgaaacatttacgtcgccgaccgggccatatgacagcggagcattactggtaa |
| Condon Optimised *Albidovulum xiamenense* CYP116B64 Reductase (+ natural linker predicted by THMM (2))  NCBI: WP_092497994.1 | gagagagcggtatcattcccgatcggagcgcctgtaaaggagaacatgatcctaaggcgtgtcgtaatcgtggaagcaagggaagaggcagagggagtaaagagcttcctattagccgacccgcgtggaaggcccttaccggggtggagcgcgggagcccacgtagaccttgtcagtggcgggttcagaaggaagtacagtctttgctccgcaccgggtgaagagggaaggtggagaatagcaatacttcgtgaggccgagggtaggggtggatcgaggcacttctgcgacgccttatccgagggcgccgaggtccaggtagcggggcctcgtaatcacttcaggcttgacgagagtgcagccaggtacaggttaatcgccggtggaataggaataacgcccatattagcaatggccgacaggttaaaggtcctgggcagggactacaccttacactactgcggacctagcagggcaaggatggccttcttagacagggtgctagccgaccacggcccgcgtgcacagttacacgtgagtgacgaggggacccgtctagaccttgcaggcgaattcgccgcagtaaccgagggtgaacaggtgtacgcatgcggccctgcgaggatgctagacgccttaagggagcttgcccgtggttggcccgaaggcgcactgcacttcgagtacttcacaacaggcgcagcaacgttagaccctgccagggagcacgcattcgaggccgtacttgccgactcaggacttgtggtagaggtcgcggcagacgagaccctttacgcggcattaagaagggccggaatcgacatccagtcagactgcggtgagggattatgcggcagctgcgaggccagggtggtagagggtgagatcgaccaccgtgacagggtattatccagggcagagagggcgagaggggacaggatgatgacgtgctgcagccgtgccaaggggcgtaggatcgtcctgggcttatga |

Additional Table 4. **List of guide RNA sequences used in this study**

| **Locus/Name** | **Guide Sequence** | **Source** |
| --- | --- | --- |
| ARS 416d | TAGTGCACTTACCCCACGTT | (5) |
| ARS 511b | CAGTGTATGCCAGTCAGCCA | (5) |
| X2_F | GACTTTTATTTAAAGAGTGCACCTTG | (6) |
| X2_R | AACAAGGTGCACTCTTTAAATACAAA | (6) |

Additional Table 5. **List of linkers used in this study**

| **Number** | **Name** | **Sequence** | **Source** |
| --- | --- | --- | --- |
| L1 | *Taxus* *cuspidata* CYP725A4- Reductase Linker | DVSTEQSAKEAPAETLGAFR | (7) |
| L2 | *Taxus* *cuspidata* CYP725A4-fldA Linker | ASGAGGSEGGGSEGGTSGAT | (8) |
| L3 | fldA-Fpr Linker | EPPPP-LPPPP-LPPPP-EPPPP | (9) |
| L4 | Potential *Albidovulum xiamenense* P450 reductase | ERAVSFPIGAPVKEN | This Study |
| L5 | Additional RhFRED Linker | STHMRLASTHM | (10) |
| L6 | Natural RhFRED Linker | LHRHQPVTIGEPAAR | (10) |
| L7 | Natural P450_BM3_ Linker | SPSTEQSAKKVRKKA | (11) |

Additional Table 6. **Augmented definitive screening designs for optimising P450-reductase expressions, including controls**

| **Hemin (µM)** | **ALA (mM)** | **FAD (mM)** | **FMN (mM)** | **Riboflavin (mM)** |
| --- | --- | --- | --- | --- |
| 0 | 0 | 0 | 0 | 0 |
| 10 | 0.1 | 5 | 10 | 0.1 |
| 10 | 3 | 5 | 5 | 2 |
| 100 | 1.55 | 10 | 10 | 2 |
| 100 | 0.1 | 10 | 10 | 0.1 |
| 100 | 3 | 10 | 5 | 2 |
| 55 | 0.1 | 10 | 5 | 0.1 |
| 55 | 1.55 | 7.5 | 7.5 | 1.05 |
| 100 | 0.1 | 5 | 7.5 | 2 |
| 100 | 0.1 | 5 | 5 | 1.05 |
| 10 | 3 | 10 | 7.5 | 0.1 |
| 10 | 0.1 | 7.5 | 10 | 2 |
| 100 | 3 | 5 | 10 | 0.1 |
| 10 | 1.55 | 5 | 5 | 0.1 |
| 10 | 0.1 | 10 | 5 | 2 |
| 100 | 3 | 7.5 | 5 | 0.1 |
| 55 | 3 | 5 | 10 | 2 |
| 10 | 3 | 10 | 10 | 1.05 |
| 0 | 0 | 0 | 0 | 0 |
| 5 | 3 | 8 | 8 | 1 |
| 5 | 3 | 8 | 1 | 4.5 |
| 50 | 1.55 | 8 | 8 | 8 |
| 5 | 0.1 | 8 | 8 | 8 |
| 50 | 3 | 8 | 1 | 8 |
| 50 | 0.1 | 8 | 4.5 | 1 |
| 27.5 | 0.1 | 8 | 1 | 1 |
| 50 | 3 | 4.5 | 8 | 1 |
| 5 | 0.1 | 4.5 | 1 | 8 |
| 27.5 | 1.55 | 4.5 | 4.5 | 4.5 |
| 5 | 1.55 | 1 | 1 | 1 |
| 50 | 3 | 1 | 1 | 1 |
| 50 | 0.1 | 1 | 8 | 4.5 |
| 5 | 3 | 1 | 4.5 | 8 |
| 50 | 0.1 | 1 | 1 | 8 |
| 5 | 0.1 | 1 | 8 | 1 |
| 27.5 | 3 | 1 | 8 | 8 |

Additional Table 7. **Linear model formulas for response factors in definitive screening designs**

| **Response** | **Formula** |
| --- | --- |
| OCT | ~$5.3253 + (-1.9931\times ALA) + (0.8422\times FAD) + (-1.1021\times Riboflavin) + (10.5527\times ALA\times ALA) + (16.4958\times FAD\times FAD) + (0.2619\times ALA\times Riboflavin)$; adjusted R-square: 0.493, Predicted Accuracy: 69.38% |
| iso-OCT | ~$10.378538+ (-3.519595\times ALA) + (0.465395\times FAD) + (-1.250850\times Riboflavin) + (-0.078938\times Hemin) + (11.017660\times ALA\times ALA) + (19.611188\times FAD\times FAD) + (0.337446\times ALA\times Riboflavin) + (0.035891\times ALA\times Hemin)$; adjusted R-square: 0.60, Predicted Accuracy: 76.78% |
| T5α-ol | ~$11.280421+ (-3.963745\times ALA) + (0.546135\times FAD) + (-1.422747\times Riboflavin) + (-0.086131\times Hemin) + (10.783645\times ALA\times ALA) + (22.517707\times FAD\times FAD) + (0.039468\times ALA\times Hemin) + (0.381168\times ALA\times Riboflavin)$; adjusted R-square: 0.63, Predicted Accuracy: 77.95% |
| OD_600_ | ~$15.1228 + (-2.0453\times ALA) + (1.0456\times FAD) + (-2.1826 \times Riboflavin)$; adjusted R-square: 0.598, Predicted Accuracy: 76.96% |
| ROS | ~$2.18395 + (-0.42513\times FAD) + (2.07776 \times Riboflavin) + (-1.89673 \times FAD\times Riboflavin)$; adjusted R-square: 0.779, Predicted Accuracy: 80.92% |


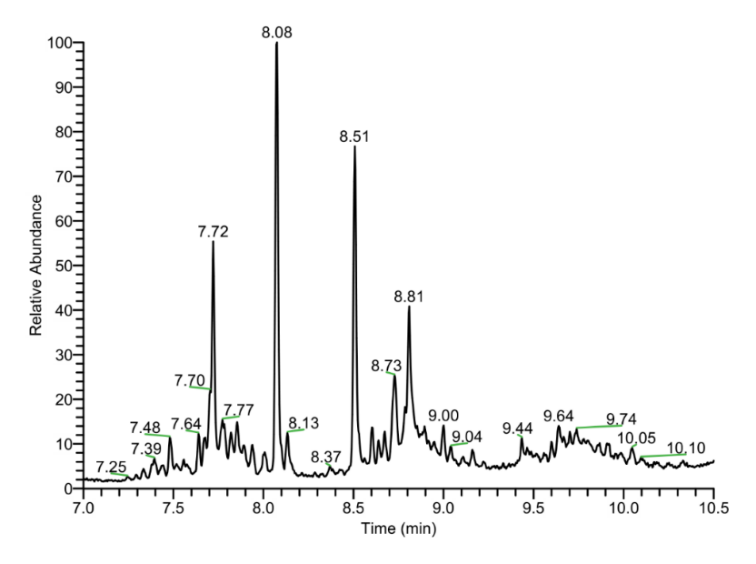


Additional Figure 1. **The messy chromatogram from 2 mL of LRS6 strain culture in polypropylene-made deepwell microplates.** It depicts the incompatibility of dodecane overlay with polypropylene material and unreliability of final results.

**
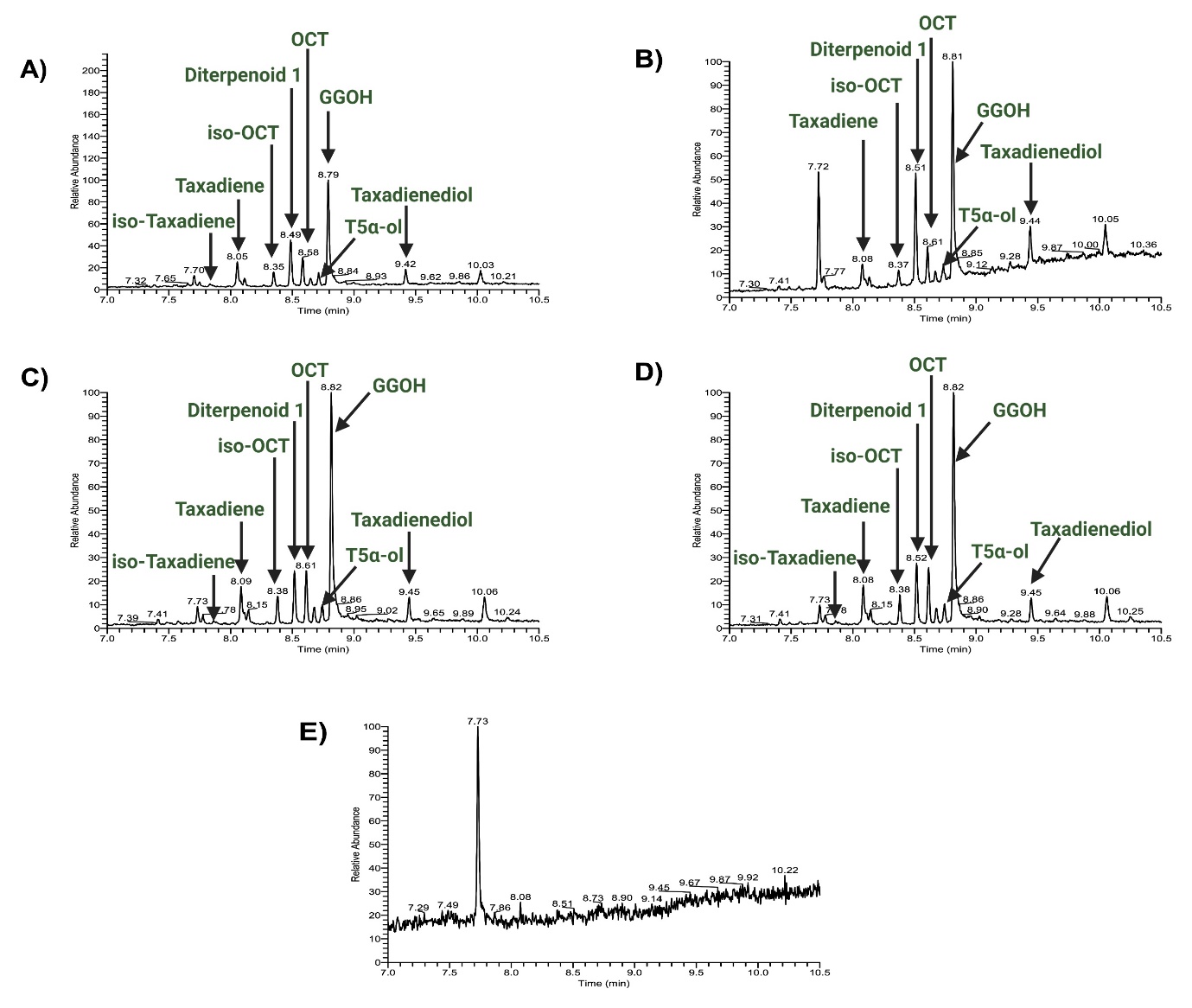
**

Additional Figure 2. **Representative chromatograms of gene dosage study.** A) LRS6; B) BN1; C) BN2; D) BN3; E) BN4

Additional Figure 3. **Representative mass spectrum for LRS6 strain cultivated in shake flask.** A) 7.38-Verticilline; B) 7.75-Diterpene 1; C) 7.83- iso-Taxadiene; D) 8.05- Taxadiene; E) 8.11- Diterpene 2; F) 8.35- iso-OCT; G) 8.48- Diterpenoid I; H) 8.58-OCT; I) 8.72- T5α-ol; J) 8.79- GGOH; K) 9.41-Taxadienediol; L) 10.02-Diterpenoid 2


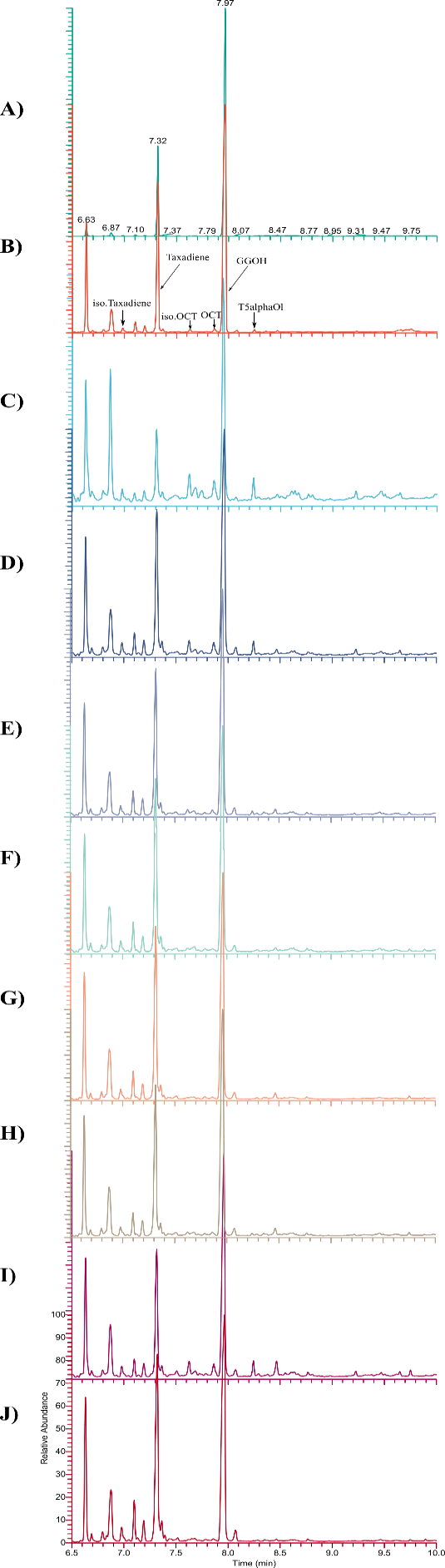


Additional Figure 4. **Representative chromatograms of the strains expressing self-sufficient CYP725A4, CYP725A4 only and parent taxadiene-producing LRS5, all cultivated in 5 mL of YPG in 10 mL glass tubes.** A) LRS5; B) BN5; C) BNF-1; D) BNF-2; E) BNF-3; F) BNF-4; G) BNF-5; H) BNF-6; I) BNF-7; J) BNF-8


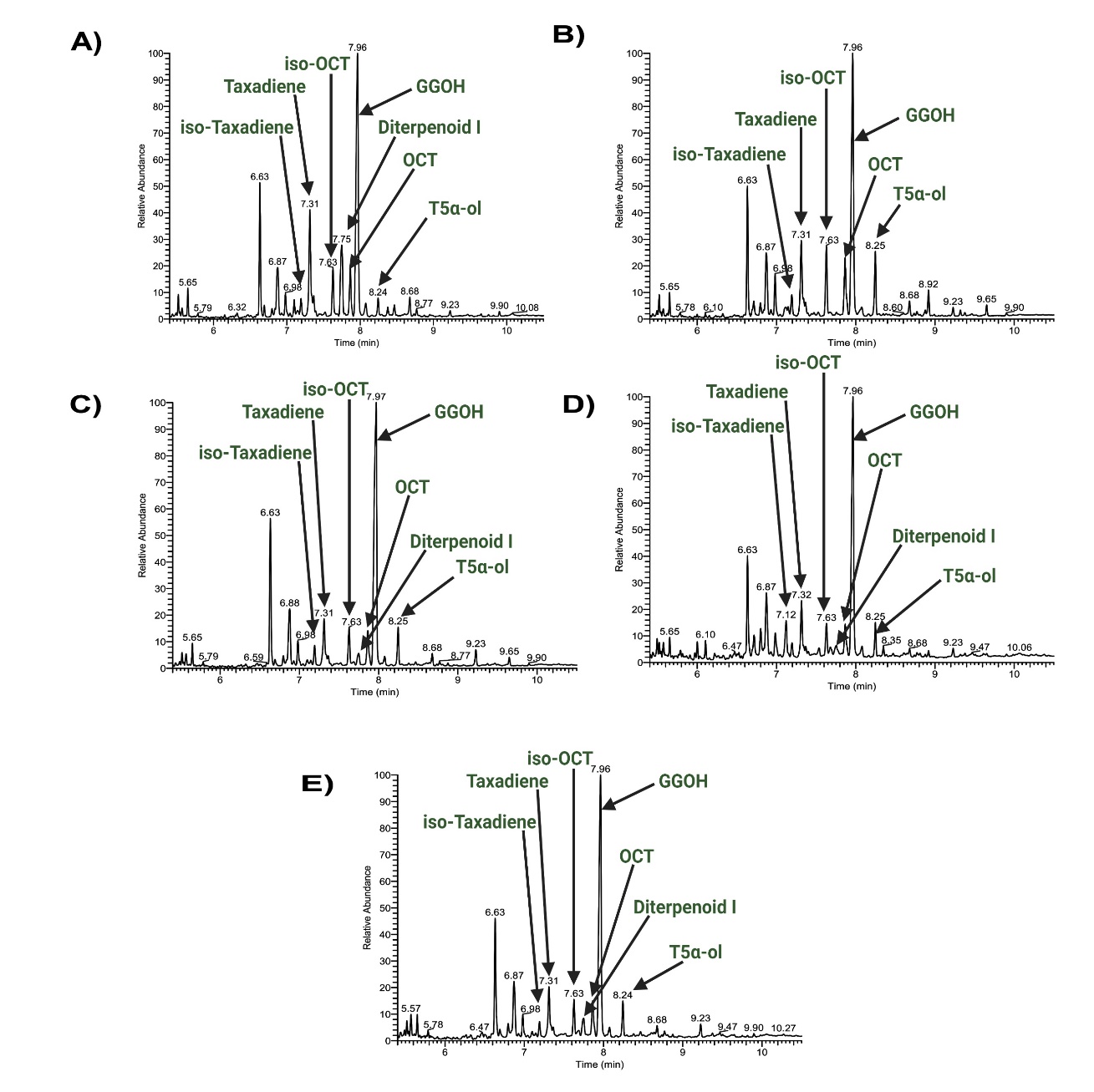


Additional Figure 5. **Representative chromatograms for BN6, expressing Taxus CYP725A4 and POR in tandem, grown in different culture media.** A) TBMG; B) SDG; C) BHIG; D) LBG; E) YPG. BHIG: Brain Heart Infusion Broth-2% (w/v) galactose; LBG: LB Broth (Lennox)- 2% (w/v) galactose; SDG: Synthetic Defined-2% (w/v); YPG: Yeast Extract Peptone-2% (w/v) galactose; TBMG: Terrific Broth, Modified-2% (w/v) galactose.


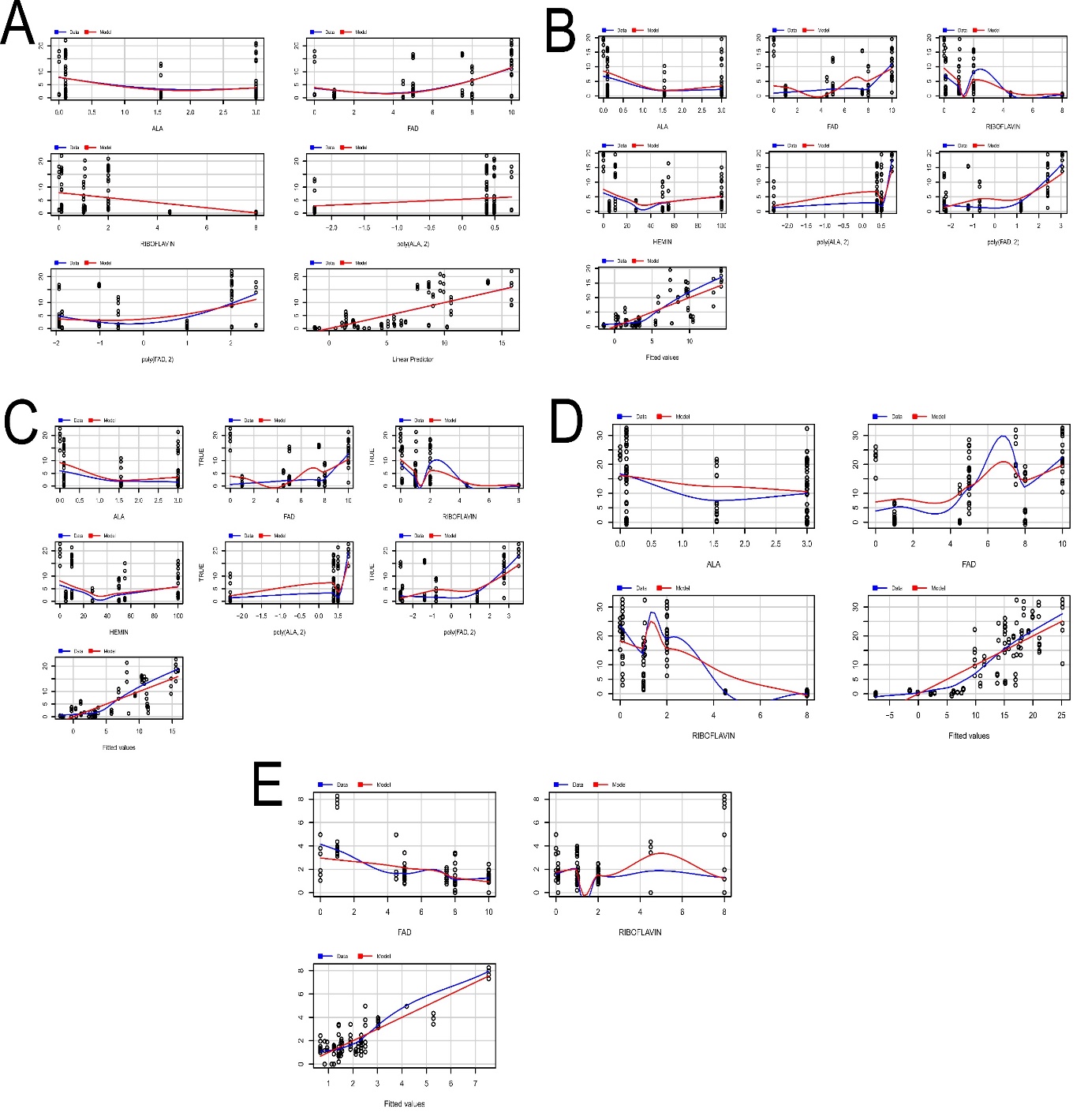


Additional Figure 6. **Marginal model plots for design of experiment (DoE) study for all predictors (Flavin Adenine Dinucleotide (FAD), Flavin Mononucleotide (FMN), Riboflavin, Hemin and δ-Aminolevulinic acid (ALA)) and their interactions on** A) OCT; B) iso-OCT; C) T5α-ol; D) OD_600_; E) Reactive Oxygen Species (ROS) responses.


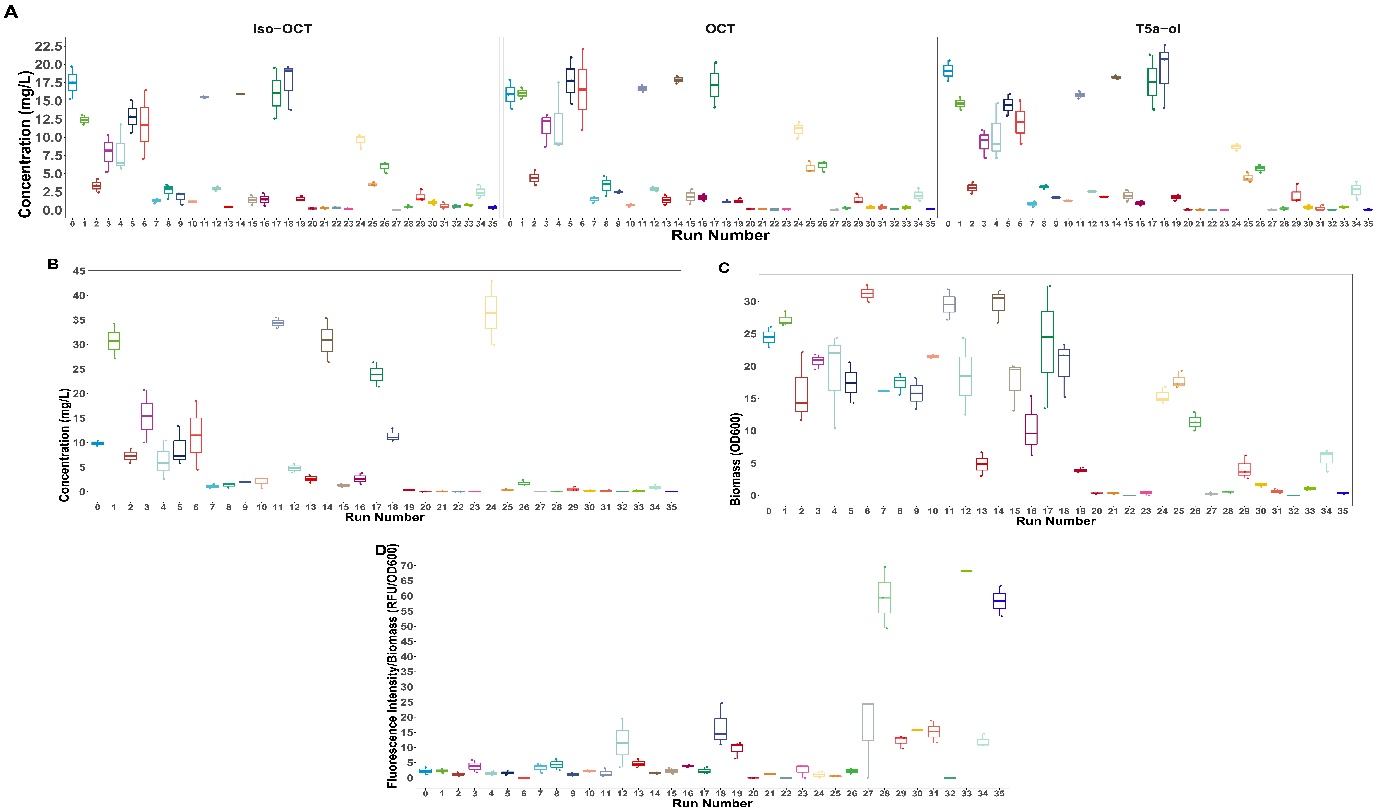


Additional Figure 7. **DoE study results according to run number.** A) iso-OCT, OCT and T5α-ol; B) Taxadiene; C) Biomass (OD_600_); D) Relative amount of ROS normalised by final OD_600_. Values represent means ± SD of triplicate samples.


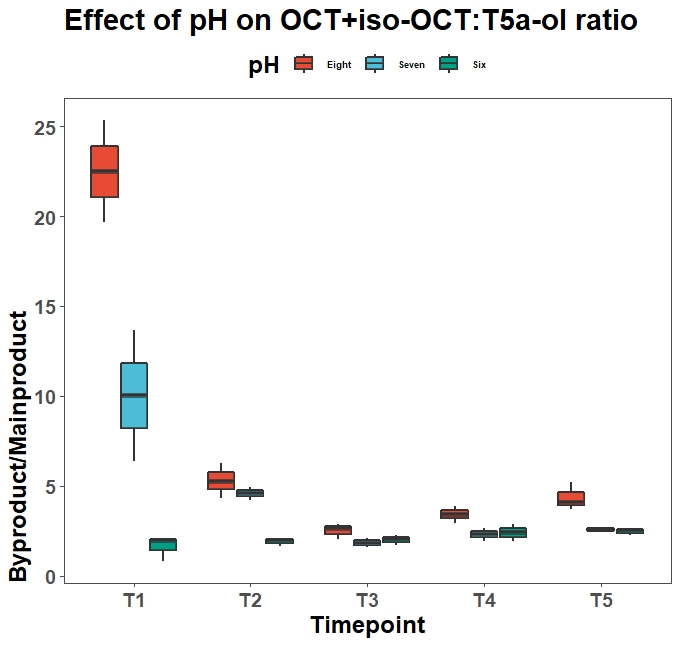


Additional Figure 8. **Preliminary resting cell assay to test the effect of acid-base on side-product (OCT+ iso-OCT) to main product (T5α-ol) ratio.** The experiment was performed in 10 mL glass tubes, under microaerobic condition, using 50 mL of phosphate buffer with pH 6, 7 and 8, supplemented with 20 g/L galactose during five days with starting OD_600_= 40. Values indicate means ± SD of triplicate samples. T1: 21 hours; T2: 28 hours; T3: 50; T4: 98 hours; T5: 115 hours.


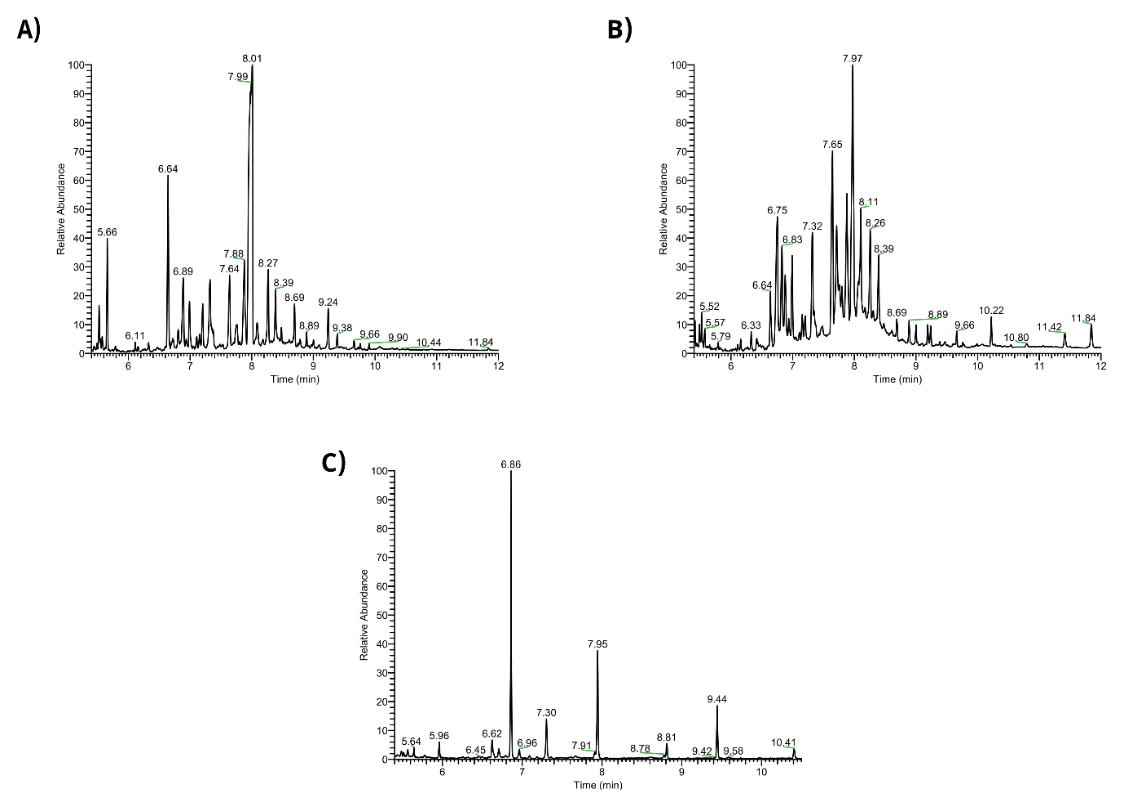


Additional Figure 9. **Representative chromatograms for the resting cell assays.** A) Galactose induction during growth stage; B) Galactose induction during the resting stage only; C) Parallel run with LRS5 to relate the new peaks to Taxus CYP725A4- Taxus POR expression in BN6 denote that the oxygenated taxanes were formed due to monooxygenase activity. The iso-OCT, OCT and T5α-ol eluted at approximately 7.64, 7.88 and 8.26 minutes.


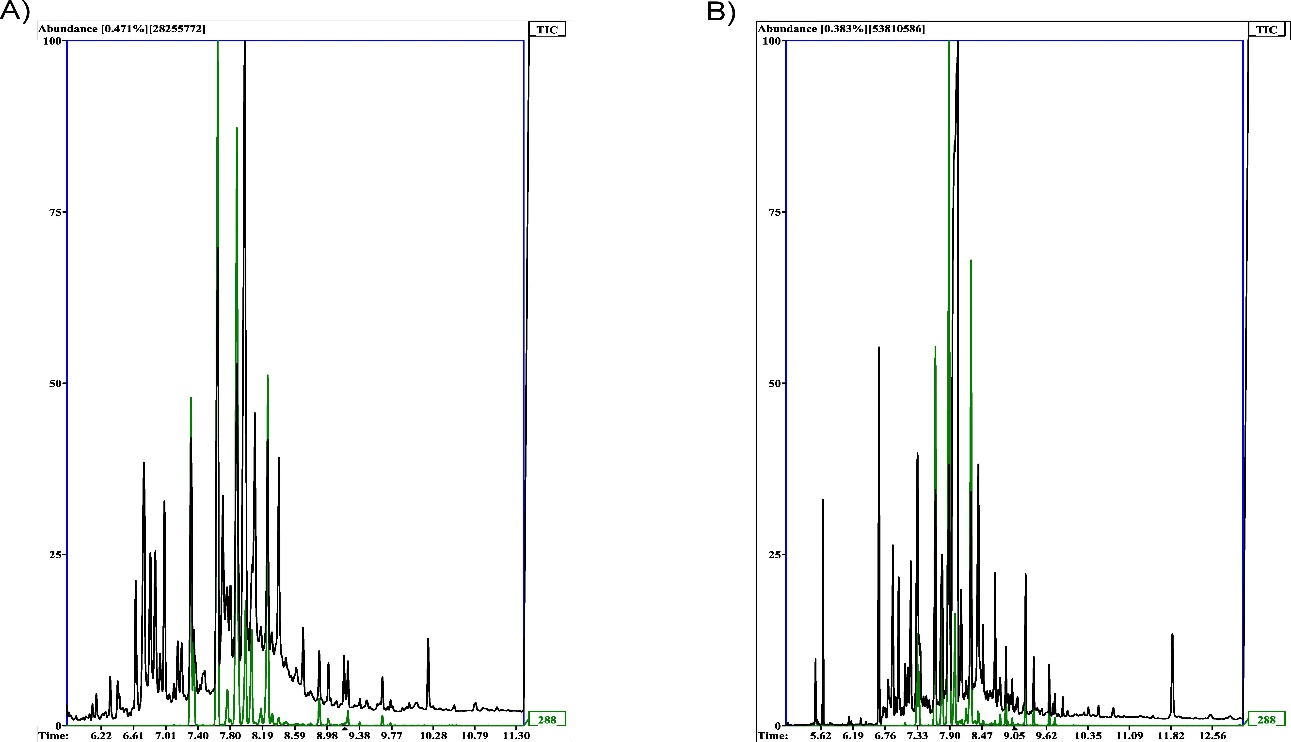


Additional Figure 10. **Representative chromatograms for the resting cell assays with extracted ion of 288 m/z for confirming the identified diterpenoids.** A) Galactose induction during resting stage; B) Galactose induction during the growth stage only. The analysis was performed by AMDIS software.

Additional Figure 11. **Representative mass spectra from resting cell assay experiment.** Identified terpenes and terpenoids are as follows: Q) 7.10-iso-Taxadiene; S) 7.19-Nerolidol; T) 7.34-Oxygenated Taxadiene; U) 7.37-Diterpenoid II; V) 7.62-iso-OCT; W) 7.74-Diterpenoid I


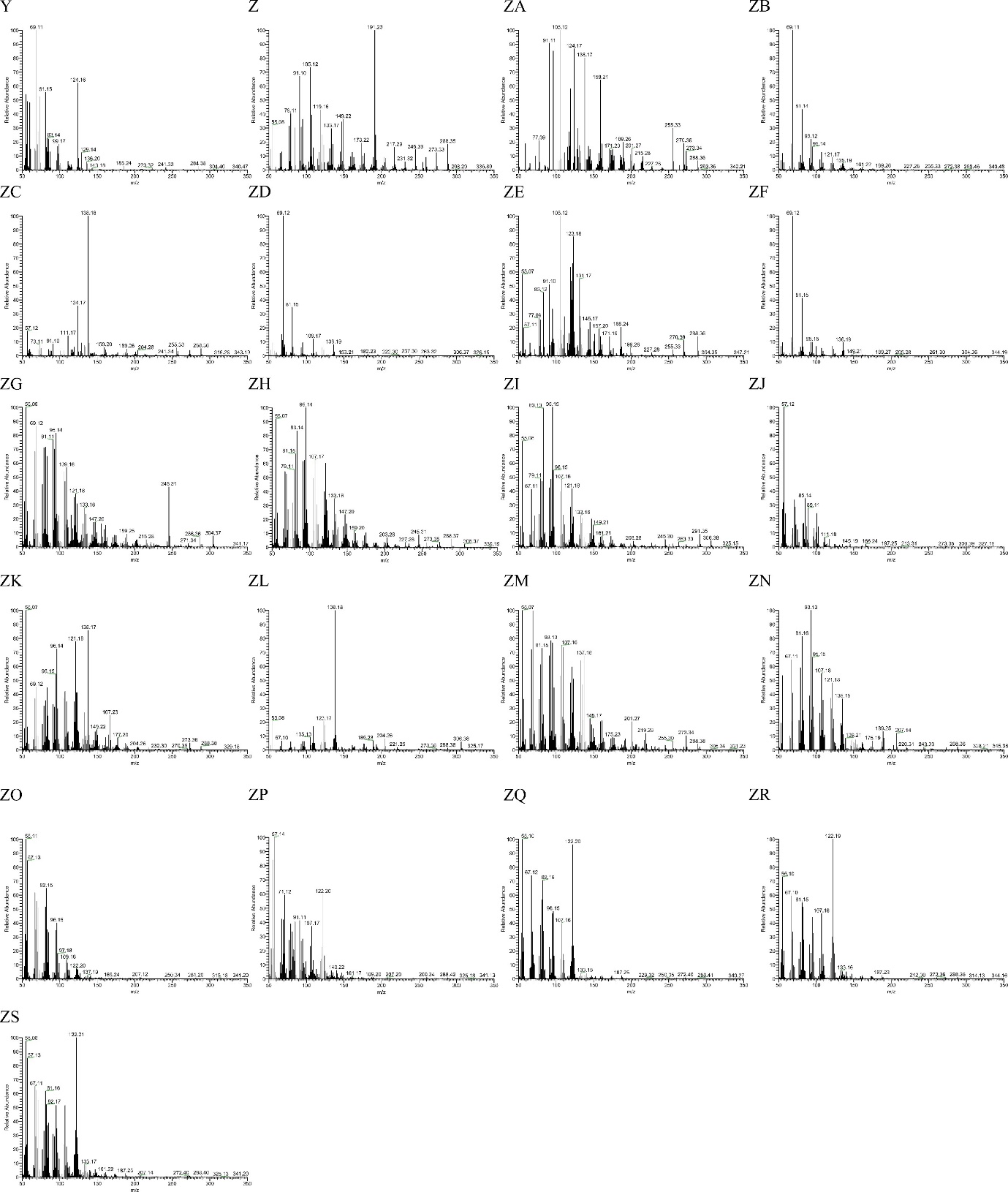


Additional Figure 12. **Representative mass spectra from resting cell assay experiment.** Identified terpenes and terpenoids are as follows: Z) 7.86-OCT; ZA) 7.89-Diterpenoid U with co-elution with OCT; ZB) 7.94-GGOH; ZC) 8.05-Diterpenoid III; ZE) 8.24- T5α-ol; ZG) 8.67-Taxadienediol; ZH) 8.88- Diterpenoid IV; ZI) 8.99-Diterpenoid V; ZK) 9.23-Diterpenoid VI; ZL) 9.38-Diterpenoid VII; ZM) 9.65-Diterpenoid VIII; ZN) 9.75-Diterpenoid IX; ZO-S) 7.34-Taxadiene/Oxygenated Taxadiene peak during pre-resting galactose induction experiment from time 1 to time 5. The ion 306 m/z in ZH, ZI, ZL and ZM might represent the inclusion of H_2_O relative to 288 m/z ion. The peaks of 286 and 304 m/z in ZG denote that it is taxadienediol (1).

References

1. Walls LE, Malcı K, Nowrouzi B, Li RA, D’Espaux L, Wong J, et al. Optimizing the biosynthesis of oxygenated and acetylated Taxol precursors in *Saccharomyces cerevisiae* using advanced bioprocessing strategies. Biotechnol Bioeng [Internet]. 2020 Sep 16;118(n/a):279–93. Available from: https://doi.org/10.1002/bit.27569

2. Krogh A, Larsson B, von Heijne G, Sonnhammer EL. Predicting transmembrane protein topology with a hidden Markov model: application to complete genomes. J Mol Biol. 2001 Jan;305(3):567–80.

3. Omura K, Aiba Y, Onoda H, Stanfield JK, Ariyasu S, Sugimoto H, et al. Reconstitution of full-length P450BM3 with an artificial metal complex by utilising the transpeptidase Sortase A. Chem Commun [Internet]. 2018;54(57):7892–5. Available from: http://dx.doi.org/10.1039/C8CC02760A

4. Kanehisa M, Goto S. KEGG: kyoto encyclopedia of genes and genomes. Nucleic Acids Res. 2000 Jan;28(1):27–30.

5. Reider Apel A, d’Espaux L, Wehrs M, Sachs D, Li RA, Tong GJ, et al. A Cas9-based toolkit to program gene expression in *Saccharomyces cerevisiae*. Nucleic Acids Res [Internet]. 2016/11/24. 2017 Jan 9;45(1):496–508. Available from: https://www.ncbi.nlm.nih.gov/pubmed/27899650

6. Mikkelsen MD, Buron LD, Salomonsen B, Olsen CE, Hansen BG, Mortensen UH, et al. Microbial production of indolylglucosinolate through engineering of a multi-gene pathway in a versatile yeast expression platform. Metab Eng [Internet]. 2012;14(2):104–11. Available from: https://www.sciencedirect.com/science/article/pii/S1096717612000079

7. Johnson EO, Wong L-L. Partial fusion of a cytochrome P450 system by carboxy-terminal attachment of putidaredoxin reductase to P450cam (CYP101A1). Catal Sci Technol [Internet]. 2016/09/01. 2016 Oct 21;6(20):7549–60. Available from: https://pubmed.ncbi.nlm.nih.gov/28944003

8. Wang Q, Huang X, Zhang J, Lu X, Li S, Li J-J. Engineering self-sufficient aldehyde deformylating oxygenases fused to alternative electron transfer systems for efficient conversion of aldehydes into alkanes. Chem Commun [Internet]. 2014;50(33):4299–301. Available from: http://dx.doi.org/10.1039/C4CC00591K

9. Bakkes PJ, Riehm JL, Sagadin T, Rühlmann A, Schubert P, Biemann S, et al. Engineering of versatile redox partner fusions that support monooxygenase activity of functionally diverse cytochrome P450s. Sci Rep [Internet]. 2017;7(1):9570. Available from: https://doi.org/10.1038/s41598-017-10075-w

10. Robin A, Roberts GA, Kisch J, Sabbadin F, Grogan G, Bruce N, et al. Engineering and improvement of the efficiency of a chimeric [P450cam-RhFRed reductase domain] enzyme. Chem Commun [Internet]. 2009;(18):2478–80. Available from: http://dx.doi.org/10.1039/B901716J

11. Govindaraj S, Poulos TL. Probing the structure of the linker connecting the reductase and heme domains of cytochrome P450BM-3 using site-directed mutagenesis. Protein Sci [Internet]. 1996 Jul;5(7):1389–93. Available from: https://pubmed.ncbi.nlm.nih.gov/8819171
